# Transcriptome Sequencing in Response to Salicylic Acid in *Salvia miltiorrhiza*

**DOI:** 10.1371/journal.pone.0147849

**Published:** 2016-01-25

**Authors:** Xiaoru Zhang, Juane Dong, Hailong Liu, Jiao Wang, Yuexin Qi, Zongsuo Liang

**Affiliations:** 1 College of Life Sciences, Northwest Agriculture & Forestry University, Yangling, Shaanxi, People's Republic of China; 2 College of Life Sciences, Zhejiang Sci-Tech University, Hangzhou, Zhejiang, People's Republic of China; University of Western Sydney, AUSTRALIA

## Abstract

*Salvia miltiorrhiza* is a traditional Chinese herbal medicine, whose quality and yield are often affected by diseases and environmental stresses during its growing season. Salicylic acid (SA) plays a significant role in plants responding to biotic and abiotic stresses, but the involved regulatory factors and their signaling mechanisms are largely unknown. In order to identify the genes involved in SA signaling, the RNA sequencing (RNA-seq) strategy was employed to evaluate the transcriptional profiles in *S*. *miltiorrhiza* cell cultures. A total of 50,778 unigenes were assembled, in which 5,316 unigenes were differentially expressed among 0-, 2-, and 8-h SA induction. The up-regulated genes were mainly involved in stimulus response and multi-organism process. A core set of candidate novel genes coding SA signaling component proteins was identified. Many transcription factors (e.g., WRKY, bHLH and GRAS) and genes involved in hormone signal transduction were differentially expressed in response to SA induction. Detailed analysis revealed that genes associated with defense signaling, such as antioxidant system genes, cytochrome P450s and ATP-binding cassette transporters, were significantly overexpressed, which can be used as genetic tools to investigate disease resistance. Our transcriptome analysis will help understand SA signaling and its mechanism of defense systems in *S*. *miltiorrhiza*.

## Introduction

*Salvia miltiorrhiza* Bunge is one of the perennial herbs that is widely cultivated in East Asia. As a famous traditional Chinese herbal medicine, its dried roots and rhizomes are used as medicinal parts to treat cardiovascular and cerebrovascular diseases, hyperlipidemia and acute ischemic stroke [1–3]. Both lipid-souble tanshinones, such as tanshinone I, tanshinone IIA, tanshinone IIB, cryptotanshinone, and water-soluble phenolic acids, including rosmarinic acid and salvianolic acids, are bioactive components that exhibit antioxidant, antitumor, anti-inflammatory and antibacterial functions [2,4]. However, the growth, yield and quality of *S*. *miltiorrhiza* are influenced by diseases, insect pests and environmental stresses, such as drought, salinity and high or low temperature.

Salicylic acid (SA), a simple phenolic compound existed widely in higher plants, not only regulates plant growth and metabolism, but also plays a leading role in plant immunity against diseases and environmental stresses, such as salt, cold and heavy metals [5–8]. Exogenous supply of SA can stimulate transcription of pathogenesis related (PR) genes and the development of systemic acquired resistance (SAR) in *Arabidopsis thaliana*, and enhance plant resistance [9]. Blocking SA accumulation through mutation or application of inhibitor of SA biosynthesis-related enzymes enhanced the susceptibility to pathogen, yet the resistance can be restored through exogenous SA [10]. Lots of studies have provided insights into the SA signaling in plant immunity, of which many main components have been identified. In SA signaling, nonexpressor of pathogenesis_related protein 1 (NPR1) is a master regulator that interacts with downstream transcription factors (TFs) and control the expression of PR genes in multiple immune responses, including SAR [11]. NPR4 and NPR3 are two SA receptors that sense the SA gradient and regulate NPR1 level during biotic and abiotic stresses [12]. NIM interacting protein (NIMIN) is another NPR1-interacting protein that negtively regulates PR gene expression [13]. At the downstream of SA signaling, TGA is a key NPR1-activated regulatory TF family, which targets glutathione S-transferases (GSTs) and PRs that involve in detoxification and defense [14–16]. WRKY TF family was also reported to act on downstream of NPR1 mediating SA signaling [17]. More recent studies demonstrated that Mitogen activated protein kinase (MAPK) signaling cascade was also involved in SA signaling system [18–20]. Although SA plays such an important role in plant immune system and so many studies on the SA signal transduction has been reported in other plants, the SA signaling pathway remained largely unknown in *S*. *miltiorrhiza*.

Second generation sequencing technology, also called RNA sequencing (RNA-seq), is powerful for gene identification, comparative gene expression analysis and investigation of functional complexity of transcriptome [21]. In recent years, RNA-seq approach has been widely used in Chinese herbal medicine for novel genes identification and differentially expressed genes (DEGs) analysis owing to its characteristics of “high throughput, low cost, covering a multitude of low abundance gene sequencing depth, and high sensitivity” [21–25]. *S*. *miltiorrhiza* is a potential model plant in the traditional Chinese medicine research field. Several transcriptome analysis projects have been performed to determine the biosynthetic processes of bioactive compounds in different *S*. *miltiorrhiza* tissues [21,25] or response to different induction [23,24]. However, to date, no systematic expression analysis of defense resistance in *S*. *miltiorrhiza* is available, and expression analyses of SA signaling-related genes in immunity are rare.

Therefore, we detected the transcriptional profiles of *S*. *miltiorrhiza* cell cultures in response to SA induction using an Illumina HiSeq 2500. The RNA-seq data generated a mass of gene resources of *S*. *miltiorrhiza*, and provided an opportunity for comprehensive understanding of biological process induced by SA. A number of genes associated with defense signaling were identified, which can be used as genetic tools to investigate disease resistance. A core set of candidate novel genes coding SA signaling components have also been identified. Further researches on these identified genes will help understanding and exploring the molecular mechanisms and genetic modulation of SA in mediating defense and stress response in *S*. *miltiorrhiza*.

## Materials and Methods

### Plant materials and sample preparation

Seeds of *S*. *miltiorrhiza* were provided by Tasly Plant Pharmaceutical Co., Ltd. (Shangluo, China). The establishment of suspension culture cell lines and SA elicitation treatment followed the methods described in our previous study [6]. Two gram fresh weight (FW) calli cells were inoculated in 50 ml Erlenmeyer flasks and cultured for 6 days, followed by SA induction. Leaf calli cells under 22.5 mg L^-1^SA induction for 0 h, 2 h and 8 h were frozen immediately in liquid nitrogen after harvest, and stored at—80°C for use. Two replicates at 0 h post induction (hpi) and three independent replicates at 2 hpi and 8 hpi were collected, respectively (each replicate from individual Erlenmeyer flask).

### RNA-seq and library construction

Total RNA was isolated using Trizol (Invitrogen, Carlsbad, CA, USA) and treated with RNase-free Dnase I (TaKaRa, Japan) for removing DNA contamination. The RNA integrity was assessed by Agilent 2100 Bioanalyzer (Santa Clara, CA, USA). The RNA-seq and construction of the libraries were performed by the Biomarker Biotechnology Corporation (Beijing, China) and the cDNA library was sequenced using Illumina HiSeq^TM^2500 with PE100. The generated sequence dataset were submitted to the National Center for Biotechnology Information (NCBI) in the Short Read Archive (SRA) database under accession number SRX1423774.

### De novo transcriptome assembly and functional annotation

In order to obtain the clean reads, the raw reads were fitered by removing the adapter, poly-N and low quality sequences. De novo assembly was performed using the Trinity method [26]. The K-mer and group pairs distance were set at 25 and 300, respectively, while the other parameters were set at default levels. Based on their overlap regions, the short reads were assembled into longer contigs, which were then clustered and further assembled into unigenes with the paired-end information.

Unigenes were aligned to a series of protein databases using Blastx (E-value ≤ 10^−5^), including the NR, Swiss-Prot, Gene Ontology (GO), Cluster of Orthologous Groups of Proteins (COG), Kyoto Encyclopedia of Genes and Genomes (KEGG) databases. The open reading frames (ORFs) were predicted by the Getorf program.

### Quantification of gene expression

The clean data were mapped to the unigene library using Bowtie [27]. Then, the read count for each gene was obtained from the mapping results by RSEM [28]. FPKM [29] for each unigene was calculated to determine the unigene expression profiles. Differential expression analysis of any two sample groups were analyzed using DESeq [30] with Benjamini and Hochberg False Discovery Rate (FDR) method [31]. And here, the FDR < 0.01 and Fold Change (FC) ≥ 2 or ≤ -2 were set as the threshold to identify DEGs. Cluster analysis was performed according to the patterns of unigene differential expression across the samples.

### q PCR validation

Total RNAs were extracted from *S*. *miltiorrhiza* leaf calli cell cultures and treated with RNase-free Dnase I (TaKaRa). The reverse transcription reaction was performed by using SuperScript III (RT kit; Invitrogen) following the manufacturer’s recommendations. qPCR analysis was carried out on the IQ5 Mul-ticolor Real-Time PCR Detection System (BIO-RAD, Hercules, CA) using SYBR Green PCR Master Mix (Vazyme, Nanjing, China). Each reaction contained 10 μl 2× SYBR Green Master Mix Reagent (Vazyme), 2 μl of cDNA sample and 0.4 μl of gene-specific primers. The total volume was 20 μl. The cycling conditions were: 95°C for 10 min, followed by 40 cycles of 95°C for 5 s and then 59°C for 30 s. The primers for each unigene were designed on Primer 5 software (S1 Table). *SmACTB* was used as internal control. The relative expression levels were calculated by the 2^-△△CT^ method [32].

### Determination of reduced glutathione (GSH)

The GSH was extracted from 1 g FW finely ground calli by 5 mmol·L^-1^ EDTA-TCA and to a constant volume of 9 mL. The reaction mixture contained 0.4 mL 1 mol·L^-1^ NaOH, 1.5 mL 0.1 mol L^-1^ K_3_PO_4_ buffer, 0.1 mL 4mmol L^-1^TDNB and 2 mL homogenate (pH 6.5–7.0), reacting at room temperature for 5 min, then to a constant volume of 5 mL using distilled water. The absorbance was determined at 412 nm and the GSH content was calculated according to the following equation:

$$GSHcontent\;\left(\mu g/g(FW)\right)=\frac{C\times V_{t}}{V_{S}\times F_{w}}$$

(where *C*: GSH concentration of sample obtained from standard curve calculation; *Vt*: total volume of extracts; *Vs*: the volume of the extraction liquid when the extraction is determined; *F*_*W*_: fresh weight of sample.)

### Measurement of superoxide dismutase (SOD) and peroxidase (POD) enzyme activities

SOD activity was determined by measuring its ability to inhibit the auto-oxidation of pyrogallol as described previously [6]. The SOD was extracted at 4°C from 1 g FW finely ground calli by 10 mL of a pre-cooled solution of 1.33 mM diethylene-triamine penta acetic acid in 50 mM of potassium phosphate buffer (pH 7.8). After the homogenate was centrifuged twice at 4°C for 15 min at 27 000 rpm, the supernatant was retained for the SOD assay. The reaction mixture contained 1 mL of 0.6 mM pyrogallol, 1.5 mL of 100 mM Tris–HCl buffer (pH 8.2), 0.5 mL of 6 mM EDTA and 0.1 mL of enzyme extract. The rate of pyrogallol auto-oxidation was measured from the increase in absorbance at 420 nm in a spectrophotometer after an interval of 15 s up to 2 min. One unit of SOD activity was defined as the amount of enzyme that would inhibit 50% of pyrogallol auto-oxidation.

POD activity was measured by monitoring the increase in absorbance at 470 nm in 50 mM of phosphate buffer (pH 5.5) containing 1 mM of guaiacol, 0.5 mM of H_2_O_2_ and 0.1 mL of enzyme extract. One unit of POD activity was defined as the amount of enzyme that caused an increase in absorbance of 0.01 of material per min.

### Isozyme analysis

For enzyme determination, 1 g FW of calli was homogenized in 8 ml pre-cooled 0.02 mol·l^-1^ PBS (including 1% PVP). After the homogenate was centrifuged at 10,000 rpm for 10 min at 4°C, the supernatant was retained for isozyme analysis [33].

Vertical PAGE was used to separate isozyme for analysis. Stacking gel’s concentration was 3% and the separation gel’s was 6%. The eletrode buffer was Tris-Gly (pH = 8.3). Gels were run at constant current of 8 mA at stacking phase and 15 mA at separation phase at 4°C. Staining procedures of SOD and POD were in accordance with nitro-blue tetrazolium method and acetic acid-amine method [34]. The electrophoretograms were analyzed with the number of bands, relative mobility (R_*f*_), and staining intensity. The results were recorded by digital camera.

### Statistics

Statistical analysis was carried out by using the analysis of variance (ANOVA) and SPSS 19.0 software. Differences were separated out by using the *t*-test at a 0.05 level.

## Results and Discussion

### Illumina sequencing and de novo assembly

To gain a comprehensive overview of the transcriptional response of *S*. *miltiorrhiza* to SA induction, we carried out a transcriptomic analysis of *S*. *miltiorrhiza* cell cultures with SA induction for 0 h, 2 h and 8 h, respectively. To enhance data stability, the biological repeats of induced samples were also prepared and their cDNA were produced. Eight libraries, including two 0 hpi libraries (T1 and T2), three 2 hpi libraries (T3, T4 and T5) and three 8 hpi libraries (T6, T7 and T8), were sequenced using an Illumina HiSeq™ 2500 with the production of about 100 bp paired-end reads.

After stringent data filtering and quality assessment, 16 930 116 and 16 304 238 clean paired-end reads from T1 and T2, 17 092 890, 16 519 814 and 14 846 248 clean paired-end reads from T3, T4 and T5, and 17 074 504, 15 929 201 and 15 182 299 clean paired-end reads from T6, T7 and T8 were generated (S2 Table). The Q30 percentages (percentage of sequences with sequencing error rates <1‰), and GC percents were also illustrated in S2 Table, which showed that the Q30 percentages of these samples were not less than 86.65% (S2 Table).

**Fig 1:**
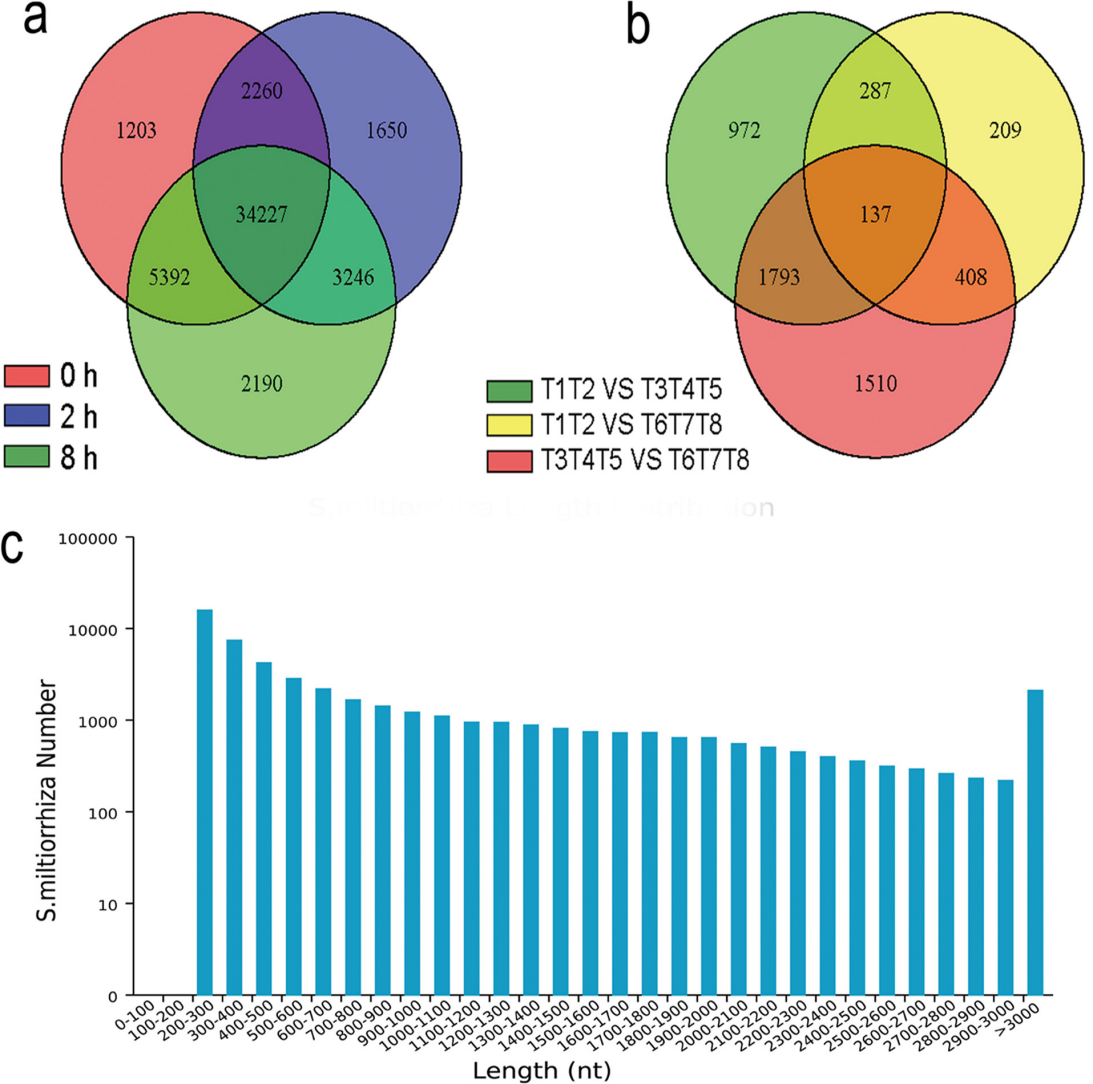
mRNA profiling of SA induced *S*. *miltiorrhiza* cell cultures by RNA-seq. (a) The common and unique expression profiles among sample groups. Numbers represent expressed unigenes in control (0 h) and SA (2 h and 8 h) treated cell cultures. (b) Number of DEGs found among different sample groups, according to a FDR< 0.01 and FC ≥ 2 or ≤ -2. T1, T2 belong to control group, T3, T4 and T5 belong to treatment group of SA induction for 2 h, T6, T7 and T8 belong to treatment group of SA induction for 8 h. (c) Length distribution of the 50 778 assembled unigenes (digital details see S3 Table).

Then, all the high quality clean reads, corresponding to 26.22 Gb clean data from the eight libraries, were assembled by Trinity program [26]. We acquired 125 024 transcripts and 50 778 unigenes, with N50 of 2 105 bp and 1 618 bp and mean lengths of 1350.06 bp and 868.75 bp, respectively (Fig 1A and 1C, S3 Table). The assembly showed high integrity. The length distribution of these generated unigenes was showed in Fig 1C. There were 27 528 unigenes (54.21%) shorter than 500 bp, 9341 unigenes (18.40%) between 500 and 1000 bp, 8203 unigenes (16.15%) between 1000 bp and 2000 bp, and 5706 unigenes (11.24%) longer than 2000 bp (S3 Table). In addition, ORFs were predicted by Getorf and 50 469 unigenes (99.39%) had ORFs with a start codon (S1 Fig).

### Unigene annotation and functional classification

The entire unigenes were aligned to the NR, Swiss-Prot, GO, COG, KEGG databases using Blastx with E-value less than 1E-5 to investigate their functions. Among these 50 778 unigenes, 24 181 (47.62%) were annotated (S4 Table), but the rest 26597 were not documented. It may be due to the technical limitation, such as read length and sequencing depth or the specificity of *S*. *miltiorrhiza* genes to some extent [35].

**Fig 2:**
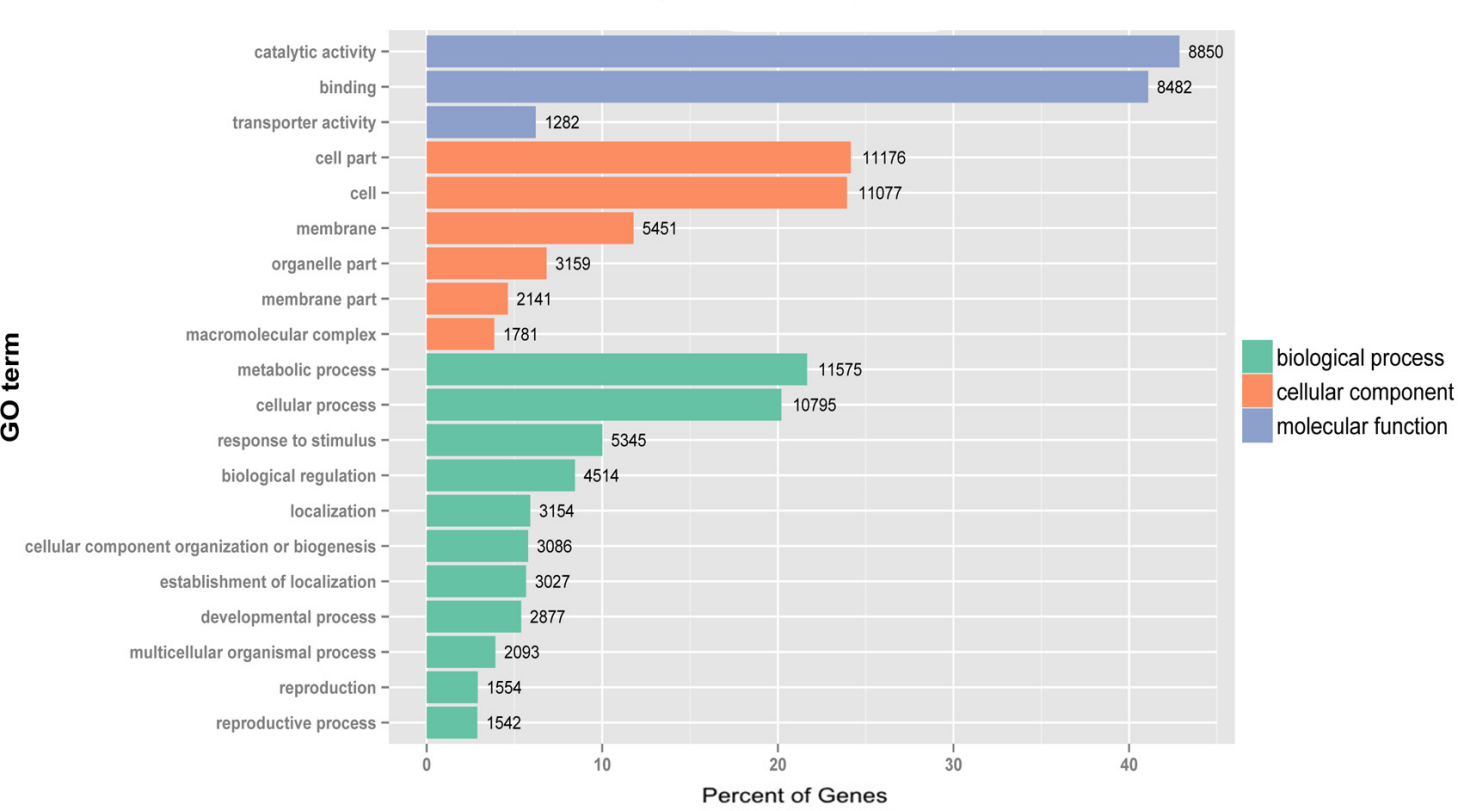
The most enriched GO terms (level 2) in unigenes of *S*. *miltiorrhiza* cell cultures. All 17 867 unigenes predominantly belonged to ‘Catalytic activity’ and ‘Binding’ under Molecular function, ‘Cell part’ and ‘Cell’ under Cellular component, and ‘Metabolic process’ and ‘Cellular process’ under Biological process. The number of unigenes belonging to each category are provided.

Cellular component, molecular function and biological process GO terms were assigned for unigenes to categorize their functions. A total of 17 867 unigenes (29.28%) were assigned to at least one GO term. This categorization generated 25926 assignments to cellular component, 27 108 assignments to molecular function and 52 782 assignments to biological process (S2 Fig). The assignments were enriched in the ‘Cell’ (GO:0005623), ‘Cell part’ (GO:0044464), ‘Binding’ (GO:0005488), ‘Catalytic activity’ (GO:0003824), ‘Cellular process’ (GO:0009987) and ‘Metabolic process’ (GO:0008152) (Fig 2). The majority of GO assignments of unigenes generated were consistent with the previous transcriptome studies of *S*. *miltiorrhiza* [23,25], which instructed the high reliability of our data.

To detect the unigenes involved in which biochemical pathway, the pathway analysis based on Blastx against the KEGG database was performed. All 4960 unigenes (9.77%) were annotated to 137 metabolic pathways (S3 Fig). The KEGG annotation information of all these sequences can help us better understand the biological function of these obtained unigenes.

### DEG analysis and validation by qPCR analysis

The r (Pearson’s correlation coefficient) [36] among biological repeat samples can evaluate the quality of the data and the rationality of samples selected. The results showed that r^2^ exceeded 0.91 for these repeat samples of 0, 2 and 8 hpi (S5 Table), which indicated the quality of our RNA-seq data is sufficient for subsequent DEG analysis.

**Fig 3:**
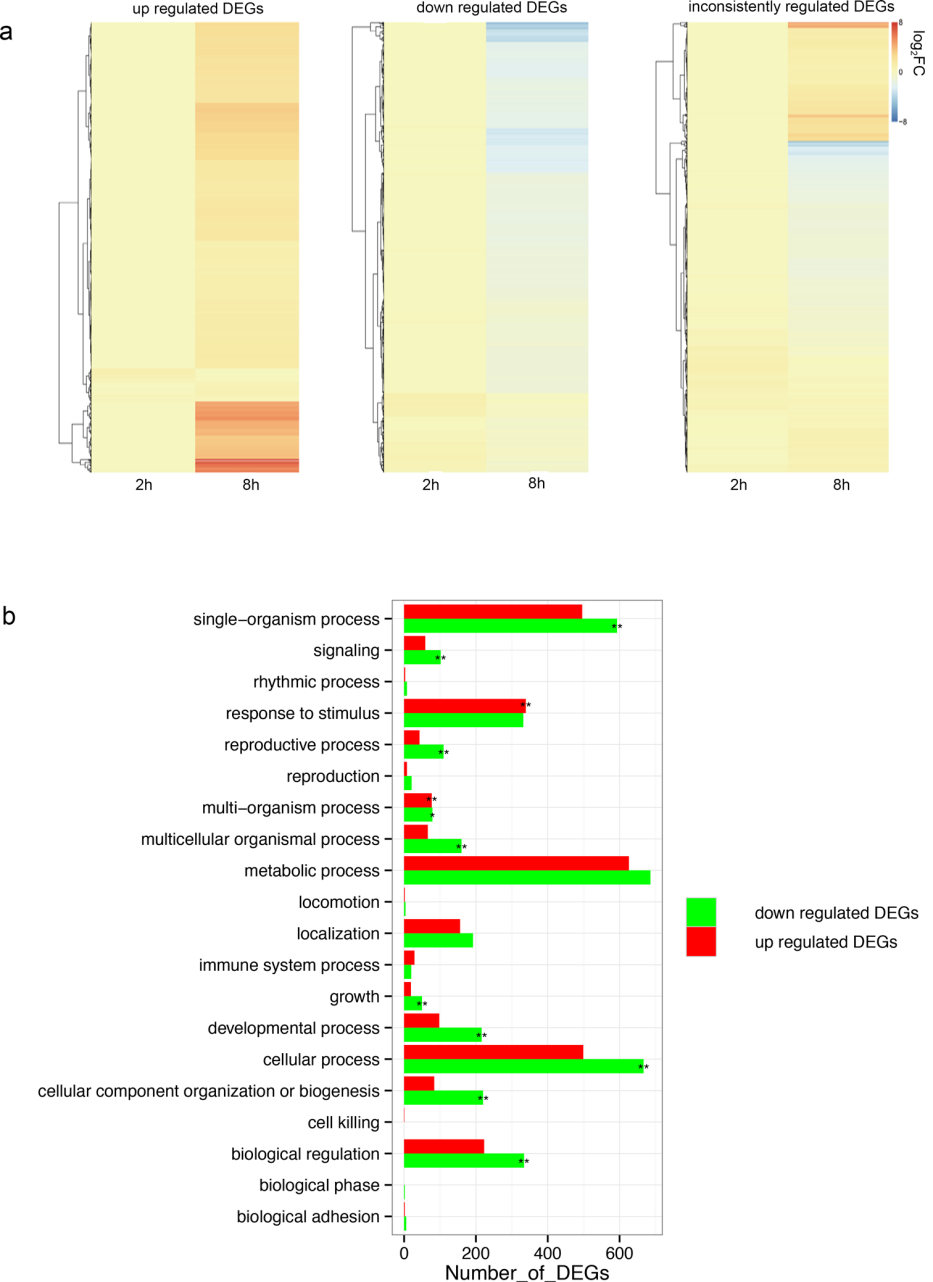
Functional analysis of DEGs in *S*. *miltiorrhiza* cell cultures after SA induction. (a) Hierarchically clustered heat map for the expression profile of DEGs (reflected as log_2_ FC when compared to control), which consist of 1584 up-regulated (left), 1492 down-regulated (middle) and 2240 inconsistently regulated DEGs (right) after 8h SA induction. Blue represent repression, whereas red represent induction. (b) Analysis of biological process category of DEGs including up-regulated (red) and down-regulated (green) in *S*. *miltiorrhiza* cells after 8h SA induction. Enrichment was measured by comparing the number of DEGs from each category with the total number of genes for that GO term and using Fisher’s exact test. Significance indicated *p*-values below 0.01 or between 0.01 and 0.05, respectively.

FPKM [29] was calculated to determine the expression levels of these unigenes. DESeq [30] was used to obtain DEGs with a FDR< 0.01 and FC ≥ 2 or ≤ -2 as cutoffs. A total of 5316 DEGs were generated, which included 3189, 1041 and 3848 unigenes differentially expressed in response to SA induction for comparing 2 h/0 h, 8 h/0 h and 2 h/8 h, respectively (Fig 1B, S6 Table). We further grouped the 5316 DEGs into three categories according to their relative expression profiles following induction, which generated 1584 up-regulated, 1492 down-regulated and 2240 inconsistently regulated unigenes (Fig 3A, S7 Table).

**Fig 4:**
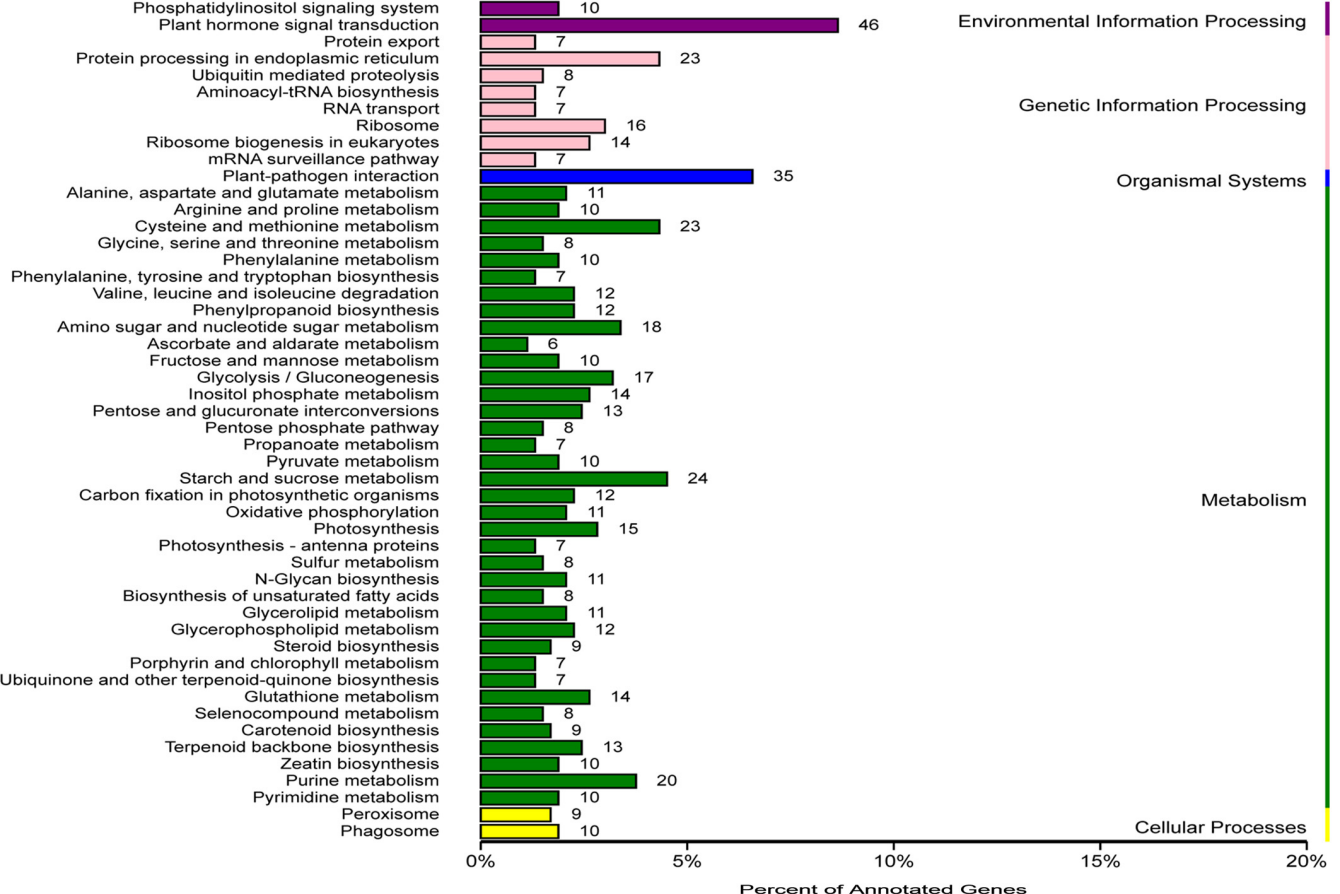
KEGG classifications of the DEGs in *S*. *miltiorrhiza* cell cultures under SA induction. A total of 532 DEGs were assigned to 104 KEGG pathways. The DEGs predominantly belonged to ‘Plant hormone signal transduction’ and ‘Plant-pathogen interaction’. The number of DEGs belonging to each category are provided.

To investigate the functions of all these 5316 DEGs, we conducted a GO analysis of all DEGs. The GO terms of ‘oxidation-reduction process’, ‘protein phosphorylation’, ‘metabolic process’, ‘response to chitin’, ‘response to cadmium ion’ and ‘response to salt stress’ were highly enriched within the biological process category (S8 Table). Most of genes categorized in molecular function were involved in ‘catalytic activity’ and ‘binding activity’ (S8 Table). ‘Cell parts’, ‘Cells’ and ‘Organelles’ were the top three categories in cell component (S8 Table). In addition, the GO analysis of up-regulated and down-regulated DEGs was also carried out. A majority of up-regulated DEGs were enriched in response to stimulus and multi-organism process, while most of down-regulated DEGs were related to the single-organism process, development and cellular process (Fig 3B). To better understand the biological pathways of these DEGs, we mapped all DEGs to terms in the KEGG database. A total of 532 DEGs were assigned to 104 KEGG pathways (Fig 4). Consistent with the results of GO analysis, the most abundant KEGG pathways in our analysis are ‘Plant hormone signal transduction’ (8.64%) and ‘Plant-pathogen interaction’ (6.58%) (Fig 4). In the ‘Plant-pathogen interaction’ pathway, the candidate genes coding Calmodulin-binding protein, WRKY and mitogen activated protein kinase kinase 5 (MKK5) were induced by SA. Some other pathways, such as the ‘Glutathione metabolism’ (2.63%), ‘Terpenoid backbone biosynthesis’ (2.44%) and ‘Phenylpropanoid biosynthesis’ (2.26%), also had a significant portion of the DEGs with pathway annotation (Fig 4). In *S*. *miltiorrhiza*, ‘Terpenoid backbone biosynthesis’ and ‘Phenylpropanoid biosynthesis’ are two main pathways involved in the synthesis of phenolic acids and tanshinones respectively, which are the main secondary metabolites. Our previous study has proved that SA induced the phenolic compounds in *S*. *miltiorrhiza* [6]. The effect of SA on these two pathways was in line with the previous study and indicated that SA may act on both the phenolic acids and tanshinones synthesis to enhance the resistance of *S*. *miltiorrhiza*. Previous research of our lab showed the H_2_O_2_ burst occurred at 2 h after SA induction in the *S*. *miltiorrhiza* cell culture [6]. In the KEGG annotion, there were 9 DEGs that annotated to the ‘peroxisome’ pathway, which indicated that the H_2_O_2_ metabolism may also associated with SA signal in *S*. *miltiorrhiza*.

Previous studies have showed that SA plays a vital role in response to disease and stress [5,8,37]. In fact, there exists complex local and systemic crosstalk among SA, reactive oxygen species (ROS, mostly in the form of H_2_O_2_) and hormone signal pathways in defense response [38–40]. These researches suggested that SA may also play a vital role in the defense response partly by interacting with ROS and other hormone signal in *S*. *miltiorrhiza*, which will be discussed in more detail in later sections. The annotation of DEGs provided a valuable resource to investigate the mechanism of SA in mediating defense responses in *S*. *miltiorrhiza*.

To verify the RNA-seq data for gene differential expression at 0, 2 and 8 hpi, the expression of 7 selected DEGs, including 1 SA-binding protein 2 (SABP2), 3 NPRs, 1 WRKY, 1 TGA and 1 POD candidate genes, were analyzed by qPCR. The trend of expression changes of these selected genes based on qPCR was similar to those detected by RNA-seq method, which corroborated the reliability and validity of the RNA-seq technology. However, the expression folds of these genes detected by qPCR had some differences with the RNA-seq data (S4 Fig). A similar situation was also found in previous study [41].

### Genes involved in SA signaling in defense response

SA signal played a critical role in triggering the defense response against biotic and abiotic stresses and activating the plant SAR [6,42,43]. The accumulation of SA up-regulated genes related to SAR in SA signaling lead to enhanced disease resistance in plants, such as tobacco and cucumber [42,44]. Given that there is no genetic resources available of SA signal in *S*. *miltiorrhiza*, we checked the expression of genes involved in SA signaling in our RNA-seq. A total of 32 candidate SA signaling-related genes were differentially expressed after SA induction, including NPR1, thioredoxins (TRXs), NPR3, NIMINs, WRKYs, TGAs, SABP2, methyl esterases (MESs) genes and several genes in MAPK cascade involved in pathogen resistance; RNA-dependent RNA polymerase 1 (RDR1) and alternative oxidase (AOX) genes involved in SA signaling against virus; and glutaredoxin (GRX) genes involved in SA- jasmine acid (JA) crosstalk (Table 1).

**Table 1:** Fold change at each time point of candidate genes involved in SA signaling in *S*. *miltiorrhiza* under SA induction in RNA-seq.

			Log_2_FC	
Unigene ID	Predicted function	Gene ID (swissprot/nr)	at 2 hpi	at 8 hpi
**c33824.graph_c0**	NPR1 (*Arabidopsis thaliana*)	sp|P93002|NPR1_ARATH	0.72	2.08
**c36545.graph_c0**	TRX H-type 1 (*Nicotiana tabacum*)	sp|P29449|TRXH1_TOBAC	1.64	1.38
**c28236.graph_c0**	TRX-like (*Arabidopsis thaliana*)	sp|Q8VZT6|TRL32_ARATH	2.43	0.35
**c23618.graph_c0**	TRX-like (*Arabidopsis thaliana*)	sp|Q9ZUU2|AAED1_ARATH	1.08	0.55
**c27090.graph_c0**	TRX H2 (*Arabidopsis thaliana*)	sp|Q38879|TRXH2_ARATH	1.81	1.15
**c16843.graph_c0**	NPR3 (*Arabidopsis thaliana*)	sp|Q8L746|NPR3_ARATH	1.52	2.12
**c35608.graph_c0**	NPR3 (*Arabidopsis thaliana*)	sp|Q8L746|NPR3_ARATH	0.43	0.46
**c18996.graph_c0**	NIMIN2c (*Nicotiana tabacum*)	gi|116490059|gb|ABJ98930.1|	-0.13	2.86
**c24383.graph_c0**	NIMIN-3 (*Nicotiana tabacum*)	sp|Q9FNZ4|NIMI3_ARATH	4.97	6.38
**c31850.graph_c0**	WRKY50 (*Arabidopsis thaliana*)	sp|Q8VWQ5|WRK50_ARATH	2.43	3.96
**c36603.graph_c0**	WRKY75 (*Arabidopsis thaliana*)	sp|Q9FYA2|WRK75_ARATH	1.65	0.31
**c31440.graph_c0**	WRKY70 (*Arabidopsis thaliana*)	sp|Q9LY00|WRK70_ARATH	3.49	2.7
**c32839.graph_c0**	WRKY18 (*Arabidopsis thaliana*)	sp|Q9C5T4|WRK18_ARATH	6.88	7.63
**c25919.graph_c1**	WRKY21 (*Arabidopsis thaliana*)	sp|O04336|WRK21_ARATH	-1.65	-0.39
**c27325.graph_c0**	WRKY17 (*Arabidopsis thaliana*)	sp|Q9SJA8|WRK17_ARATH	-1.42	-0.25
**c29604.graph_c0**	TGA-1A (*Nicotiana tabacum*)	sp|P14232|TGA1A_TOBAC	1.16	0.01
**c26759.graph_c0**	TGA5 (*Arabidopsis thaliana*)	sp|Q39163|TGA5_ARATH	1.56	0.19
**c11672.graph_c0**	TGA-2.1 (*Nicotiana tabacum*)	sp|O24160|TGA21_TOBAC	1.03	0.9
**c33952.graph_c0**	EDR1 (*Arabidopsis thaliana*)	sp|Q9FPR3|EDR1_ARATH	1.27	0.52
**c13200.graph_c1**	MKK5 (*Arabidopsis thaliana*)	sp|Q8RXG3|M2K5_ARATH	2.12	-0.3
**c30486.graph_c0**	MKK5 (*Arabidopsis thaliana*)	sp|Q8RXG3|M2K5_ARATH	2.37	-0.44
**c27615.graph_c0**	MAPK7 (*Arabidopsis thaliana*)	sp|Q39027|MPK7_ARATH	1.47	0.47
**c28990.graph_c0**	MAPK4 (*Arabidopsis thaliana*)	sp|Q39024|MPK4_ARATH	-0.63	-0.05
**c34849.graph_c0**	SABP2 (*Nicotiana tabacum*)	sp|Q6RYA0|SABP2_TOBAC	0.43	1.1
**c31368.graph_c0**	MES10 (*Arabidopsis thaliana*)	sp|Q8S9K8|MES10_ARATH	0.33	0.96
**c25679.graph_c0**	MES11 (*Arabidopsis thaliana*)	sp|Q9FW03|MES11_ARATH	0.72	0.97
**c24719.graph_c1**	AOX1 (*Nicotiana tabacum*)	sp|Q41224|AOX1_TOBAC	2.95	1.27
**c22661.graph_c0**	AOX4 (*Arabidopsis thaliana*)	sp|Q56X52|AOX4_ARATH	1.61	0.76
**c36196.graph_c0**	RDR1 (*Arabidopsis thaliana*)	sp|Q9LQV2|RDR1_ARATH	0.78	1.83
**c36610.graph_c0**	GRX-C9 (*Arabidopsis thaliana*)	sp|Q9SGP6|GRXC9_ARATH	3.76	2.65
**c15509.graph_c0**	GRX-C9 (*Arabidopsis thaliana*)	sp|Q9SGP6|GRXC9_ARATH	2.85	3.51
**c15004.graph_c0**	GRX-S9 (*Arabidopsis thaliana*)	sp|P0C291|GRXS9_ORYSJ	3.33	1.98

NPR1 is a master regulator in SA signaling pathway controlling multiple immune responses including SAR [11]. In npr1 mutant plants, SA-mediated PR gene expression and pathogen resistance were completely abolished [45]. In the inactive state, NPR1 resides in the cytoplasm as an oligomer bound by disulphide bonds. After induction, cytosolic TRX catalyse redox changes in NPR1 from oligomeric to monomeric forms, then the monomeric form of NPR1 could enter nucleus and regulate the downstream TFs, such as TGA and WRKY. It was reported that SA not only induces NPR1 expression, but also controls nuclear translocation of NPR1 catalyzed by TRX, which is essential for maintaining its function [46]. In our RNA-seq data, the transcription levels of one candidate NPR1 gene and four candidate TRX genes were increased under SA induction (Table 1), which indicated that there may exist similar regulation patten of SA effect on NPR1 activity, that is SA induced both NPR1 expression levels and NPR1 nuclear translocation in *S*. *miltiorrhiza*.

NPR3 and NPR4, NPR1 homologs, are two adaptor proteins that facilitate or block the NPR1 degradation by interacting with both NPR1 and Cullin 3-based E3 ligase at high or low SA concentrations, allowing the signaling action of NPR1 in the moderate range of SA concentration [12]. In *A*. *thaliana*, NPR3 was regarded as a negative regulator in immune responses, the npr3 mutant was shown to exhibit increased basal PR1 expression and enhance resistance to the oomycete Hyaloperonospora arabidopsidis isolate Noco [47]. Conversely, NPR4 was a positive regulator, the npr4 mutants decreased PR gene expression and compromised resistance to *Pseudomonas syringe* pv. tomato DC3000 [48]. In our RNA-seq data, the gene coding NPR4 was not discovered, which may be due to its absent or low expression in *S*. *miltiorrhiza* cells. While the transcription levels of two unigenes coding candidate NPR3s were increased in response to SA (Table 1). NIMIN is another NPR1-interacting protein that negtively regulates PR gene expression and suppression of *NtNIMIN2a* transcripts enhanced the accumulation of PR1 protein [13]. In our RNA-seq data, the expression of two candidate NIMIN genes were induced by SA (Table 1). These results indicated that the *NPR3* homologs and *NIMIN* homologs may play important roles in SA signaling by acting as NPR1 regulatory proteins that control NPR1 level in *S*. *miltiorrhiza*, making plants to fine-tune its defense against specific aggressors.

TGA is a key SA-dependent and NPR1-activated regulatory TF family that target GSTs and PRs that involved in detoxification and defense [49]. Tobacco *TGA1a* was the first identified TGA member bound to as-1 elements that mediate SA-inducible transcription [14]. Furthermore, a triple-knockout mutant tga2-1 tga5-1 tga6-1 was shown to be defective in the induction of PR genes and SAR in *A*. *thaliana*, which indicated their role in disease resistance [45]. It is noteworthy that three unigenes annotated as *TGA 1a* (*Nicotiana tabacum*), *TGA 2*.*1* (*N*. *tabacum*) and *TGA 5* (*A*. *thaliana*) were up-regulated by SA in our RNA-seq data (Table 1), which indicates that these three candidate TGA genes may play improtant roles in the NPR1-dependent SA signaling function on initiation of SA-responsive genes transcription in *S*. *miltiorrhiza*.

In addition to TGA, the WRKY TF family was also been testified to play principal positive or negative regulatory functions in SA-dependent defense responses in plants [17]. More than a half of *Arabidopsis* WRKY genes were induced or supressed when treated with SA treatment [9]. For instance, *WRKY50* and *WRKY75* serve as positive regulators of SA-mediated signaling in the activation of basal and R-mediated resistance in *A*. *thaliana* [50,51]. Some members of the WRKY TF family were reported to act downstream of NPR1 in SA signaling [17]. For example, *AtWRKY70*, acts on the downstream of *NPR1*, is a common regulatory element of SA and JA signal transduction pathway [52]. Overexpression of *AtWRKY70* could enhance the resistance of the transgenic plants and the expression of some SA induced genes [53]. *AtWRKY18* induced by SA positively modulated PR gene expression and resistance to the bacterial pathogen *Pseudomonas syringae*, and the potentiation of developmentally regulated defense responses by *AtWRKY18* is NPR1-dependent [54]. Our transcriptome analysis revealed that a number of candidate WRKY transcription factors were SA-dependent regulators (Table 1). Candidate genes coding WRKY 18, 50, 70 and 75 that were involved in defense response were significantly induced by SA (Table 1), while WRKY17 and 21 were suppressed (Table 1). The WRKY17 gene was reported to be a negative regulator of *WRKY70* [55]. These WRKY TFs are presented for the first time to be associated with plant SA-dependent defense responses, and their functions in *S*. *Miltiorrhiza* need to be futher identified.

MAPK was also reported to be involved in SA signaling system in plant immunity [18–20]. SA triggered the expression of enhanced disease resistance 1 (EDR1), a MAPKK Kinase (MAPKKK) functioned at the top of the MAPK cascade, negatively regulated SA signaling system [19]. GhMKK5 is a SA induced MAPKK protein and overexpressing GhMKK5 greatly elevated the expression of NPR1 and SA signaling system-induced PR1a and PR5 in plant [20]. In our RNA-seq data, one candidate EDR1 gene was up-regulated under SA induction, and the transcript level of 2 candidate MKK5 genes were significantly increased at 2 hpi (Table 1), which were showed to regulate expression of iron SOD gene under salinity stress [56]. We also detected a slight reduction of the transcription level of candidate MAPK4 gene in response to SA (Table 1), which has been reported to act downstream of SA and to negtively regulate SA signaling system [18]. In addition, one unigene annotated as AtMAPK7 protein, which may be involved in the transcription activition of PR1 gene acting in downstream of MKK3 in pathogen defense [57], was up-regulated by SA in our RNA-seq data (Table 1). All the response of genes involved in MAPK cascade to SA induction indicated that MAPK may also play an important role in the SA signaling system in *S*. *Miltiorrhiza*.

SA signal also plays an important role in SAR. The establishment of SAR require translocation of SA signal from the initial site of attack to the distant pathogen-free organs in SA-dependent defenses activation [58]. Owing to SA was transported upward only in very small amounts via xylem, MeSA as a mobile signal moved through phloem and was then converted to active SA form by the esterase activity of SABP2 in tobacco or members of the AtMES family in Arabidopsis in the the distant pathogen-free organs [59]. NtSABP2 is a SA-binding protein that has a strong affinity to SA in plant, and its activity was regulated by SA [60]. Silencing *NtSABP2* inhibited the local resistance to tobacco mosaic virus and reduced the expression of *PR-1* gene induced by SA, thus hindering the development of SAR [60]. Knock-down the expression of multiple AtMES genes also attenuated the SAR [61]. In our RNA-seq data, one SA responsive unigene, c34849.graph_c0, coding *NtSABP2* homolog was identified, and it was induced by SA induction at 8 hpi. The expression of two unigenes coding AtMES10 and AtMES11 homologs were increased under SA induction (Table 1), which indicated that the proteins encoded by these three genes may also be MeSA esterase that participates in the SA signal transduction in immune response of *S*. *miltiorrhiza*.

It was suggested that SA signal inducing resistance against viruses may be different from those known resistance pathways, such as NPR1-dependent pathway [62]. Mitochondrial signaling processes was reported to regulate some aspects of SA-induced virus resistance [63]. AOX functioned in the mitochondrial signaling processes and positively regulated SA-induced resistance to a tobamovirus, Turnip vein clearing virus (TVCV) [64]. Small RNA-directed RNA silencing is another potent immune surveillance system against viral pathogens [65]. The RDR1 was implicated in small RNA-directed RNA silencing and antiviral defense, and was also induced by SA treatment and virus infection [66]. In our RNA-seq data, two candidate AOX genes and one candidate RDR1 gene were up-regulated (Table 1), which suggested SA signal may enhance the efficiency of mitochondrial signaling processes and RNA silencing pathway in triggering immune responses against viruses by activating AOX and RDR1 in *S*. *miltiorrhiza*, respectively.

### SA-responsive antioxidant genes in *S*. *miltiorrhiza*

ROS members (mostly in the form of H_2_O_2_) are typical chemical signals, and SA promoted H_2_O_2_ accumulation in the early stage of induction, keeping H_2_O_2_ content essential for defense responses in plant [43]. H_2_O_2_ is an important signal involved in adaptability signaling triggering tolerance to various abiotic and biotic stresses at low concentrations, but also directly leads to lipid peroxidation and programmed cell death at high concentrations [67]. Thus there is antioxidant system regulating H_2_O_2_ in plant cell, including antioxidases, such as POD and SOD, and non-enzymatic antioxidant, such as GSH [68,69]. In our previous study, the H_2_O_2_ burst occurred at 2 h after SA induction in the *S*. *miltiorrhiza* cell culture. However, the relevance of this to the elicitation method was uncertain. Thus we detected the genes related to H_2_O_2_ metabolism in our RNA-seq data, the generated SA-responsive candidate genes, including POD, SOD, copper chaperone for superoxide dismutase (CCS) genes and glutathione metabolism-related genes, were showed in Table 2.

**Table 2:** Fold change at each time point of candidate genes involved in H_2_O_2_ burst and GSH metabolism in *S*. *miltiorrhiza* under SA induction in RNA-seq.

			Log_2_FC	
Unigene ID	Predicted function	Gene ID (swissprot/nr)	at 2 hpi	at 8 hpi
**H**_**2**_**O**_**2**_ **burst**				
**c27388.graph_c0**	peroxidase N1 (*Nicotiana tabacum*)	sp|Q9XIV8|PERN1_TOBAC	1.47	0.40
**c26177.graph_c0**	peroxidase 40 (*Arabidopsis thaliana*)	sp|O23474|PER40_ARATH	1.40	1.03
**c34235.graph_c0**	peroxidase (*Nicotiana sylvestris*)	sp|Q02200|PERX_NICSY	1.49	0.26
**c26499.graph_c0**	SOD [Cu-Zn] (*Solidago canadensis*)	sp|O04997|SODCP_SOLCS	0.94	0.04
**c26957.graph_c0**	CCS (*Arabidopsis thaliana*)	sp|Q9LD47|CCS_ARATH	1.24	0.67
**GSH metabolism**				
**c18218.graph_c0**	GST L3 (*Arabidopsis thaliana*)	sp|Q9LZ06|GSTL3_ARATH	1.98	2.35
**c24436.graph_c0**	GST APIC (*Nicotiana tabacum*)	sp|P46440|GSTF2_TOBAC	2.57	1.61
**c24433.graph_c0**	GST F9 (*Arabidopsis thaliana*)	sp|P46440|GSTF2_TOBAC	2.66	1.26
**c30193.graph_c0**	GST 23 (*Zea mays*)	sp|Q9FQA3|GST23_MAIZE	1.64	1.39
**c36714.graph_c0**	GST (*Nicotiana tabacum*)	sp|Q03662|GSTX1_TOBAC	1.33	1.54
**c18835.graph_c0**	γ-ECS (*Solanum lycopersicum*)	sp|O22493|GSH1_SOLLC	1.35	-0.10
**c33677.graph_c0**	GS (*Solanum lycopersicum*)	sp|O22494|GSHB_SOLLC	1.40	0.72
**c21482.graph_c0**	GR(*Brassica rapa subsp*. *Pekinensis*)	sp|O04955|GSHRC_BRARP	1.91	0.62

**Fig 5:**
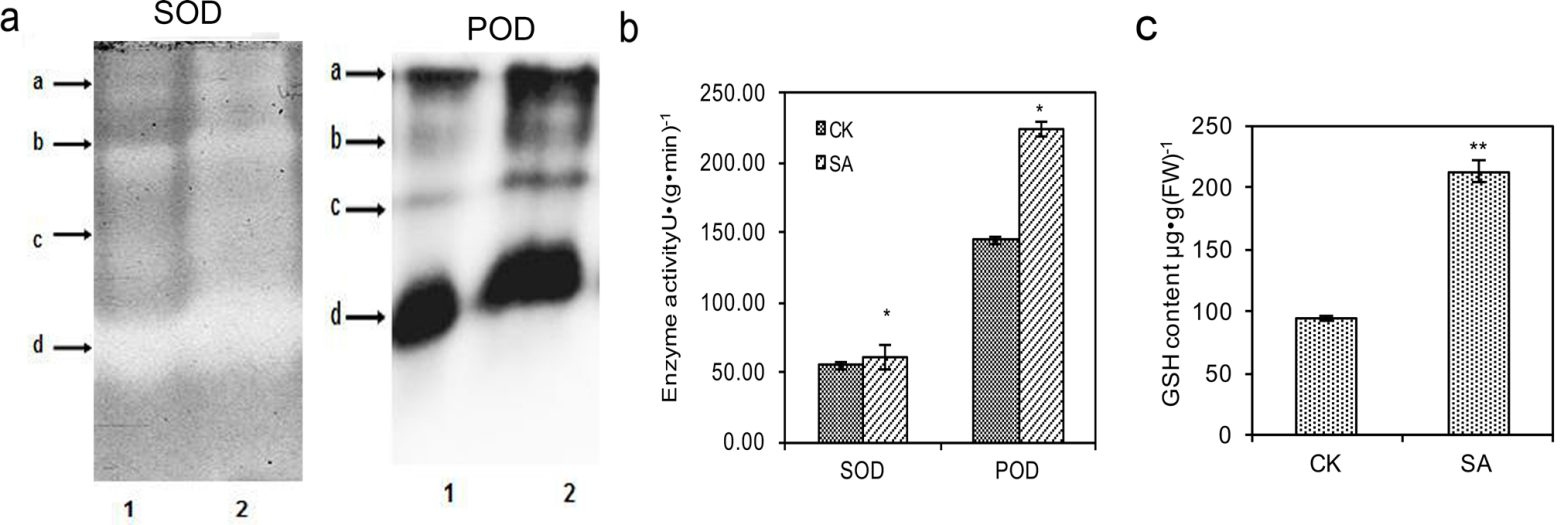
Effect of SA on antioxidative enzymes and GSH in *S*. *miltiorrhiza* cell cultures. (a) Effect of SA on isozymograms of SOD (left) and POD (right). 1 represents control and 2 represents SA treatment for 2 h. a, b, c, d represents four bands of SOD and POD, respectively. (b) Effect of SA on enzyme activities of SOD and POD. (c) Effect of SA on the content of GSH. Significance was indicated by double or single asterisks with *p*-values below 0.01 or between 0.01 and 0.05, respectively.

Of the antioxidative enzymes, the extracellular POD is one source of H_2_O_2_ [70]. SOD constitutes the first line of defense against ROS and dismutated the superoxide to produce H_2_O_2_ [71]. CCS is a helper protein that acts to insert copper and oxidize the disulfide in the maturation process for SOD in eukaryotes [72]. When expressed in *Saccharomyces cerevisiae*, Cu/Zn-SOD was activated by the *AtCCS* in *Arabidopsis thaliana* [73]. Many studies have emphasized that, SA can enhance the SOD and POD activities to protect plants from damage [74,75]. Our results also showed that three candidate POD genes, one candidate SOD gene and one candidate CCS gene were up-regulated at 2 hpi under SA induction (Table 2), which was in line with our previous study that the H_2_O_2_ burst occurred after 2-h SA induction. The up-regulation of POD, SOD and CCS genes indicated that SA enhanced the activation of Cu/Zn-SOD and transcription of POD and SOD in *S*. *miltiorrhiza*. We further examined the effect of SA on isozymograms and activities of SOD and POD (Fig 5A and 5B). As shown in Fig 5A, patterns of SOD and POD showed clear band differences after SA treatment. Four bands of each enzyme were obtained, respectively (Fig 5A). The bands of SOD and POD in SA treatment were wider and showed stronger intensity than that of the control (Fig 5A). Consistent with the isozymogram analysis, the SOD and POD activities were significantly increased by 1.11- and 1.55-fold after SA elicitation (Fig 5B). Our results indicated that the cultured cells responded SA by stimulating the antioxidative enzymes POD and SOD to protect the plant from any injuries and participate in the generation of H_2_O_2_ signal.

Of the non-enzymatic antioxidants, glutathione is one vital part of theredox hub [69]. H_2_O_2_ is reduced to H_2_O by the reaction of glutathione peroxidase with GSH, which is oxidized to GSH disulfide (GSSG), GSSG can be reduced back to GSH by GSH reductase (GR) [76]. GSH reacts with electrophilic group of endogenous and xenobiotic harmful substances mediated by GST to form mixed disulfides, and plays a critical role in cellular detoxification [77]. GSH synthesis requires two enzymes: gamma-glutamylcysteine synthetase (γ-ECS) and glutathione synthetase (GS). γ-ECS mediates the first reaction between glutamate and cysteine to form a dipeptide, γ-glutamyl-cysteine (γGluCys), which is the rate-limiting enzymatic step and in turn reacts with glycine catalyzed by GS to produce GSH [76]. In our study, a total of 70 unigenes were annotated to glutathione metabolism pathway. Among these, one candidate γ-ECS gene, one candidate GS gene and one candidate GR gene were up-regulated at 2 hpi and maintained high expression levels until 8 hpi except γ-ECS, in which the expression level had no significant change at 8 hpi (Table 2). We further detected the content of GSH under SA induction in the the *S*. *miltiorrhiza* cell culture. As expected, the content of GSH was significantly increased by 2.26-fold than that of the control after the application of SA (Fig 5C). In *A*. *thaliana*, high SA concentration was associated with higher GSH contents [78]. Our results also indicated that SA increased GSH levels and reducing power (ratio GSH/GSSG) by activating the transcriptions of candidate γ-ECS, GS and GR candidate genes. Recent study has showed that, in parallel to its antioxidant role, GSH acts independently of NPR1 to allow increased intracellular H_2_O_2_ to activate SA signaling [79]. The decrease in γ-ECS protein resulted in GSH deficiency and negatively affected disease resistance [80]. Therefore, we speculated the SA-regulated GSH may play important roles in plant resistance by acting as both antioxidant and regulatory factor of SA signaling in *S*. *miltiorrhiza* cells. Notably, SA obviously increased the transcription of five candidate GST genes invloved in glutathione metabolism in our RNA-seq data (Table 2). In addition to the detoxification function, GST have been shown to be implicated in varied stress resistances, such as pathogen attack, oxidative stress, and heavy-metal toxicity [81]. It has been emphasized that GSTs are the immediate-early SA-responsive genes [82]. This result indicated that these five GSTs may play important roles in cellular detoxification, such as ROS scavenging, and SA-mediated stress resistance. The characterization of these above genes elucidated the effect of SA on the antioxidant system in *S*. *miltiorrhiza*.

### Hormone-related genes in *S*. *miltiorrhiza* in response to SA

SA significantly affected the hormone biosynthesis and signaling pathway in plant [82–85]. However, the hormone-related genes responsed to SA were unknown in *S*. *miltiorrhiza*. In this study, a number of genes involved in hormone biosynthesis and signal transduction responsed to SA were analyzed (Table 3). Hormone crosstalk is crucial for plant defenses against pathogens and insects, in which SA, JA, and ethylene (ET) play key roles [83]. Antagonism between SA and JA signaling has been well reported in plants. In our RNA-seq data, the candidate JA biosynthesis genes allene oxide synthase (AOS), allene oxide cyclase (AOC) and 9-lipoxygenase (LOX) were all repressed by SA (Table 3), which was in line with the SA supression on JA biosynthesis genes in *A*. *thaliana* [84]. It indicated that SA may suppress JA signaling system by down-regulating the biosynthesis of JA in *S*. *miltiorrhiza*. In JA signaling pathway, MYC2 is a master positive regulator that binds to *cis*-acting elements of JA response genes. In our RNA-seq data, the expression level of one candidate MYC2 gene were decreased after SA induction (Table 3), which indicated that SA may block JA signaling by depressing MYC2 expression. Previous study showed that *Arabidopsis* GRX480 was a SA-induced GRX-C9 protein that interacted with TGA factors and suppressed JA signal [86]. In our study, the similar up-regulation of candidate GRX genes were also detected as well as their proteins (Table 1). Besides, those genes coding SA signaling components NPR1, WRKY70 and TGAs have been reported to be JA signaling repressors [52,86,87], but in our RNA-seq data, they were up-regulated (Table 1). All these results indicated the suppression of SA on JA signaling in *S*. *miltiorrhiza*. Unlike SA and JA signaling antagonism, SA and ET have been reported to work synergistically in inducing resistance [85]. In *Arabidopsis*, ET is perceived by a family of five membrane-bound receptors, namly Ethylene response1 (ETR1), Ethylene response sensor1 (ERS1), ethylene response 2 (ETR2), ethylene insensitive 4 (EIN4) and ERS2. These ET receptors are negitive regulators of ET signaling [88]. ERF1 is a TF that positively functioned downstream in ET signaling system [89]. In our RNA-seq data, the candidate genes coding ET receptors ETR2 and EIN4 were down-regulated, while the expression level of one candidate gene coding positive regulator ERF1B were increased (Table 3), which indicated that SA may activate ET signaling and enhance ET-mediated resistance in *S*. *miltiorrhiza*. In additon to JA and ET, SA also interacts with other hormones such as abscisic acid (ABA), auxin, gibberellic acid (GA) and cytokinin (CK) in effective immune responses activation [82]. The antagonistic interaction between ABA and SA signaling systems has been reported in plants, ABA suppresses inducible innate immune responses by down-regulating SA biosynthesis and SA-mediated defenses in *Arabidopsis* [90]. However, ABA synthesis in *SA induction deficient2* (*sid2*) mutants plants was decreased compared with that in wild-type plants under virulent Pst DC3000 infection, which suggests that SA may be a positive regulator of ABA levels [90]. In our RNA-seq data, the expression level of candidate genes coding ABA synthesis-related enzymes 9-cis-epoxycarotenoid dioxygenase (NCED) and alcohol dehydrogenase (ADH) were increased under SA induction (Table 3), which indicated that SA elicitor enhanced the ABA synthesis in *S*. *miltiorrhiza*. PYR1-like protein (PYL), protein phosphatase 2C (PP2C, a negative regulator) and SNF1-related protein kinase (SnRK2, a positive regulator) are three major components involved in the ABA signal perception and transduction pathway. In normal plants, PP2Cs bind to SnRK2s and dephosphorylate the SnRK2s to keep the SnRK2s in an inactive state [91]. PYL is an ABA receptor protein that perceive accumulated ABA and disrupt the interaction between the SnRK2s and PP2Cs, thus activating the SnRK2 kinases and resulting ABA signaling activation [91]. In our RNA-seq data, one ABA receptor PYL4 gene was up-regulated, while the transcript level of two candidate PP2Cs genes were decreased under SA induction (Table 3). This result indicated that SA not only enhanced the ABA synthesis but also triggered ABA signaling in *S*. *miltiorrhiza*. Plants have evolved auxin signaling repression mechanisms during pathogenesis [46]. Plants overproducing SA frequently result auxin-deficient or auxin-insensitive mutants morphological phenotypes [92]. AUXIN RESISTANT1/LIKE AUXIN RESISTANT1(AUX1/LAX) functions both in basipetal auxin transport and in acropetal transport in a phloem-based auxin transport stream. Three unigenes coding LAXs were down-regulated in our RNA-seq data (Table 3), which suggesting that SA might interfere with auxin response in *S*. *miltiorrhiza*. CK is involved in various regulatory processes throughout plant development and defense responses. In CK phosphorelay signaling system, after percept CK signal, the CK receptors autophosphorylate on a conserved His residue and relay this phosphoryl group to Arabidopsis response regulators (ARRs) via an intermediate set of histidine phosphotransfer (hpt) proteins called the Arabidopsis Hpt proteins (AHPs) [93]. There are two types of ARR transcription activators have been reported. Type-A ARRs are negative regulators of cytokinin signaling, while the type-B ARR transcription factor is positive regulators of CK signaling that trigger enhancement of defense responses [93]. Interestingly, two candidate type-A ARR9 genes (negative regulator) and one candidate AHP6 gene (positive regulator) involved in CK signaling were all overexpressed under SA induction in our RNA-seq data (Table 3), which indicated that the complex regulation of SA on CK signaling in *S*. *miltiorrhiza*. GA is an important plant growth hormone involved in plant innate immunity. GA receptor insensitive dwarf 2 (GID2) and GA-insensitive 1 (GAI1) are two key components in GA signaling pathway. GID2 is a GA receptor that positively regulate GA signaling, and GAI1 is a DELLA protein that represses almost all known GA-dependent processes [94]. In our RNA-seq data, we dected that the candidate gene coding GID2, a positive regulator, were up-regulated, while the candidate DELLA protein GAI1 gene, a negtive regulator, was down-regulated (Table 3), which indicated that GA signaling was induced by SA in *S*. *miltiorrhiza*. In a word, the effects of these positive and negative regulations on other hormone-related genes suggest that SA promotes plant resistance, partly by modulating the balance between SA-mediated and other hormone-mediated defense signaling pathways.

**Table 3:** Fold change at each time point of candidate hormone-related genes in *S*. *miltiorrhiza* under SA induction in RNA-seq.

			Log_2_FC		
Unigene ID	Predicted function	Gene ID (swissprot/nr)	at 2 hpi	at 8 hpi	Hormone role
**c37047.graph_c0**	AOS (*Arabidopsis thaliana*)	sp|Q96242|CP74A_ARATH	-1.20	-0.06	JA synthesis
**c28646.graph_c1**	AOS (*Vitis vinifera*)	gi|225428606|ref|XP_002281226.1|	-0.69	-1.27	JA synthesis
**c14695.graph_c0**	AOC (*Salvia miltiorrhiza*)	gi|377552569|gb|AFB69864.1|	-1.08	-1.02	JA synthesis
**c26418.graph_c0**	LOX5 (*Arabidopsis thaliana*)	sp|Q9LUW0|LOX5_ARATH	-1.63	-0.20	JA synthesis
**c35963.graph_c0**	LOX5 (*Arabidopsis thaliana*)	sp|Q9LUW0|LOX5_ARATH	-1.89	-1.15	JA synthesis
**c32834.graph_c0**	LOX1.5 (*Solanum tuberosum*)	sp|Q43191|LOX15_SOLTU	-0.05	-1.43	JA synthesis
**c27417.graph_c0**	MYC2 (Theobroma cacao)	gi|508703788|gb|EOX95684.1|	-1.69	-0.15	JA signaling
**c36831.graph_c0**	ERF1B (*Arabidopsis thaliana*)	sp|Q8LDC8|ERF92_ARATH	3.05	0.55	ET signaling
**c30725.graph_c0**	EIN4 (*Arabidopsis thaliana*)	sp|Q9ZTP3|EIN4_ARATH	-1.02	-0.53	ET signaling
**c29674.graph_c0**	ETR2 (*Arabidopsis thaliana*)	sp|Q0WPQ2|ETR2_ARATH	-1.90	-0.48	ET signaling
**c30435.graph_c0**	NCED (*Arabidopsis thaliana*)	sp|Q9LRR7|NCED3_ARATH	2.57	0.62	ABA synthesis
**c36534.graph_c0**	ADH (*Camellia sinensis*)	gi|308943732|gb|ADO51748.1|	1.88	1.66	ABA synthesis
**c36552.graph_c0**	ADH (*Ricinus communis*)	gi|255568816|ref|XP_002525379.1|	1.61	1.48	ABA synthesis
**c27930.graph_c0**	PYL4 (*Arabidopsis thaliana*)	sp|O80920|PYL4_ARATH	2.10	0.15	ABA signaling
**c34845.graph_c0**	PP2C protein HAB1 (*Arabidopsis thaliana*)	sp|Q9CAJ0|P2C16_ARATH	-3.78	-0.60	ABA signaling
**c21120.graph_c0**	PP2CA (*Arabidopsis thaliana*)	sp|P49598|P2C37_ARATH	-3.78	0.02	ABA signaling
**c18562.graph_c0**	ARR9 (*Arabidopsis thaliana*)	sp|O80366|ARR9_ARATH	0.30	1.15	CK signaling
**c28213.graph_c0**	ARR9 (*Arabidopsis thaliana*)	sp|O80366|ARR9_ARATH	2.18	1.23	CK signaling
**c35025.graph_c0**	AHP6 (*Arabidopsis thaliana*)	sp|Q9SSC9|AHP6_ARATH	1.07	0.28	CK signaling
**c24922.graph_c0**	LAX2 (*Medicago truncatula*)	sp|Q9FEL7|LAX2_MEDTR	-1.02	-3.12	Auxin signaling
**c28766.graph_c1**	LAX2 (*Medicago truncatula*)	sp|Q9FEL7|LAX2_MEDTR	-1.83	-0.40	Auxin signaling
**c21632.graph_c0**	LAX5 (*Medicago truncatula*)	sp|Q8L883|LAX5_MEDTR	-0.72	-2.67	Auxin signaling
**c27396.graph_c0**	GID2 (*Arabidopsis thaliana*)	sp|Q9STX3|GID2_ARATH	1.40	0.41	GA signaling
**c36872.graph_c0**	DELLA protein GAI1 (*Vitis vinifera*)	sp|Q8S4W7|GAI1_VITVI	-1.48	-0.52	GA signaling

### SA-responded TFs in *S*. *miltiorrhiza*

The regulation of gene expression occurs primarily at the transcriptional level, and the most diverse regulatory protein interacting with DNA at the transcriptional level is TF. TFs have been proved to be involved in plant growth, development, defense and stress response [95,96]. However, the TFs associated with SA signlaing in defense and stress response have not been identified in *S*. *miltiorrhiza*. In this study, we investigated the unigenes encoding TFs in our RNA-seq dataset to reveal the molecular mechanisms of events that involve TFs in *S*. *miltiorrhiza*. By comparison with the TFs in Plant TFDB, we identified a total of 1188 candidate genes encoding 56 TF families and their distributions were evaluated (Table 4, S9 Table). Then the candidate TF genes were grouped into two categories, including 67 up-regulated and 105 down-regulated candidate TF genes (Table 4, S9 Table). Among the differentially expressed TFs, the NAC family (9 genes) and GRAS family (7 genes) are the most representative TF families in the up-regulated TFs (Table 4, S9 Table). NAC and GRAS, two large plant-specific TF families, have been reported to be involved in diverse biological processes, such as defense and stress tolerance [97,98]. Sun et al. (2015) had implied that a host of ONAC genes showed to be up-regulated in rice under various abiotic (salt, drought, and cold) and biotic (fungus, bacteria, viruses and parasitic plants) stresses. And the transgenic *A*. *thaliana* plants overexpressing GRAS showed stronger tolerance to drought and salt treatments [97]. This indicated the important functions of the NAC and GRAS families in SA-mediated signal transduction and in response to abiotic and biotic stresses (Table 4). By contrast, the bHLH family (12 genes) and HD-ZIP family (7 genes) are the top two TF families among the down-regulated TFs (Table 4, S9 Table). bHLH and HD-ZIP are TF families involved in plant growth and development regulation under normal growth conditions or environmental stress [99,100]. Overexpression of *PtaHB1* (one HD-ZIP TF) in transgenic *Poplar* delayed the formation of primary xylem fiber [101]. The latest research suggested that *OsbHLH120* was a vital TF in root thickness and root length under hydroponic culture in upland rice [102]. The result indicated that bHLH and HD-ZIP family TFs may play important roles in SA-mediated regulation of plant growth and development in *S*. *miltiorrhiza*. Furthermore, many candidate genes of ERF (57 unigenes), bZIP (54 unigenes) and MYB (55 unigenes) families showed to be differentially expressed after SA induction (Table 4, S9 Table). These ERF, bZIP and MYB TF families have been identified to be involved in responses to disease and environmental stress, such as drought and salt stresses in many plants following different regulatory strategies [103–106]. Therefore, we speculate that these differentially expressed candidate TF genes of ERF, bZIP and MYB families may have conserved and important functions in the positive or negative regulation of SA mediated defense and stress response in *S*. *miltiorrhiza*. These results provided a detail information on the SA-responsive TFs in *S*. *miltiorrhiza* cells.

**Table 4:** Transcription factors in response to SA elicitation in *S*. *miltiorrhiza*.

TF family	Nunbers of unigene	Up-regulated TF genes	Down-regulated TF genes
**AP2**	16	2	2
**ARF**	28	1	2
**B3**	38	2	1
**bHLH**	99	3	12
**bZIP**	54	4	9
**C2H2**	64	4	5
**C3H**	47	0	4
**ERF**	57	5	9
**FAR1**	60	3	4
**G2-like**	21	2	0
**GATA**	27	0	5
**GRAS**	49	7	4
**HD-ZIP**	54	3	7
**HSF**	12	2	0
**LBD**	36	1	0
**MYB**	55	2	5
**NAC**	71	9	4
**MYB-related**	70	3	6
**Trihelix**	22	2	1
**WRKY**	66	8	6
**others**	242	4	19
**Total**	1188	67	105

### SA-responded and defense-related cytochrome P450 (CYP450) and ATP-binding cassette (ABC) genes in *S*. *miltiorrhiza*

CYP450 and ABC families have been reported to play important roles in defense response [107–109]. While the relationship between them and SA signal remained largely unknown in *S*. *miltiorrhiza*. Thus, we detected the expression of the CYP450 and ABC families genes under SA induction in our RNA-seq data, and the results were shown in Tables 5 and 6. CYP450, one of the biggest gene superfamilies in plant, was reported to be involved in plant resistance by taking part in lignin and glucosinolates biosynthesis, callose deposition and cell wall reinforcement, which is the primary event in the host–pathogen interaction [107]. In our RNA-seq data, a total of 278 unigenes encoding candidate CYP450s were discovered, which contained 40 up-regulated DEGs induced by SA (Table 5, S10 Table). The CYP71 clan was the most representative group of up-regulated CYPs (26 CYP genes) (Table 5). In this clan, genes from the CYP71 family accounted for almost half of up-regulated (Table 5). Two CYP450 enzymes of CYP71 family, CYP71B40v3 and CYP71B41v2, were likely to be involved in herbivore-induced volatile nitrile emission in *P*. *trichocarpa* [108]. It was also reported that eight members of CYP71 clan were probably involved in yeast extract+Ag^+^ induced tanshinone synthesis in *S*. *miltiorrhiza*, three of which belonged to CYP71 family [21]. Therefore, we hypothesize that these SA-responsive CYP71s could be associated with the SA-dependent secondary metabolism in defense response in *S*. *miltiorrhiza*.

**Table 5:** Summary of Up-egulated Candidate Cytochrome P450 genes in *S*. *miltiorrhiza*.

Clan	Family	Subfamily	Mumber
**CYP71 clan**	CYP71	CYP71A	5
		CYP71D	5
	CYP75	CYP75B	3
	CYP76	CYP76B	2
		CYP76C	1
	CYP81	CYP81D	3
		CYP81E	3
		CYP81F	2
	CYP83	CYP83B	1
	CYP89	CYP89A	1
**CYP72 clan**	CYP72	CYP72A	5
	CYP714	CYP714D	1
	CYP734	CYP734A	1
**CYP85 clan**	CYP707	CYP707A	1
	CYP725	CYP725A	1
**CYP86 clan**	CYP86	CYP86B	1
	CYP94	CYP94A	3
	CYP704	CYP704C	1

**Table 6:** Fold change at each time point of candidate only-up regulated candidate ABC transporter genes in *S*. *miltiorrhiza* under SA induction in RNA-seq.

			Log_2_FC	
unigene ID	Predicted function	Gene ID(swissprot)	at 2 hpi	at 8 hpi
**c30542.graph_c0**	ABCB4 (*Arabidopsis thaliana*)	sp|O80725|AB4B_ARATH	2.13	1.17
**c34701.graph_c0**	ABCB11 (*Arabidopsis thaliana*)	sp|Q9FWX7|AB11B_ARATH	1.45	0.37
**c34701.graph_c1**	ABCB11 (*Arabidopsis thaliana*)	sp|Q9FWX7|AB11B_ARATH	2.11	1.15
**c15625.graph_c0**	ABCB15 (*Arabidopsis thaliana*)	sp|Q9LHD1|AB15B_ARATH	1.79	2.13
**c33278.graph_c0**	ABCB25 (*Oryza sativa subsp*. *japonica*)	sp|Q9FNU2|AB25B_ORYSJ	1.63	1.21
**c34846.graph_c0**	ABCC2 (*Arabidopsis thaliana*)	sp|Q42093|AB2C_ARATH	1.27	0.08
**c27640.graph_c0**	ABCC3 (*Arabidopsis thaliana*)	sp|Q9LK64|AB3C_ARATH	3.21	1.85
**c33965.graph_c0**	ABCC10 (*Arabidopsis thaliana*)	sp|Q9LYS2|AB10C_ARATH	2.24	1.45
**c27749.graph_c0**	ABCG25 (*Arabidopsis thaliana*)	sp|Q84TH5|AB25G_ARATH	5.39	3.18

Studies have demonstrated the roles of ABC transporters for vacuolar transport in xenobiotic detoxification in plants [109]. Some ABC transporters were reported to play roles in resistance to pathogens, such as fungus and barley yellow dwarf virus [110,111]. In our study, 123 unigenes coding candidate ABC transporters were detected, which included 9 up-regulated DEGs (Table 6, S11 Table). Members from the ABC transporter B and ABC transporter C families annotated to ‘Defense mechanisms’ in COG database were enriched in the up-regulated (Table 6). In addition, one candidate gene encoding ABCG25 was also up-regulated (Table 6). ABCB family plays a dual role in polar auxin transport and drought stress tolerance in *Arabidopsis* [112]. The members of ABCC family, AtABCC1, AtABCC2 and AtABCC3, were involved in phytochelatin-mediated cadmium tolerance in Arabidopsis. For example, seedlings overexpressing AtABCC3 have an increased cadmium tolerance [113]. ABCG25 was involved in ABA transport responded to cold and heat stress [114]. Therefore, the result indicated that these up-regulated ABC transporter genes belonging to ABCB, ABCC and ABCG families may play vital roles in cellular processes such as auxin influx transport, ABA transport and detoxification in SA-mediated resistance. It will be of great interest to futher discuss the function of ABC transporters since there is no available resources of ABC transporters in *S*. *miltiorrhiza*. These results provided a valuable genetic resources of CYP450s and ABCs, which may act on downstream of SA signaling in defense resistence.

## Conclusions

*S*. *miltiorrhiza* is a potential medicinal model plant with important medicinal and economic values in the traditional Chinese medicine research. In this study, we performed the transcriptome analysis to examine the early SA responses of *S*. *miltiorrhiza* cells. A total of 50 778 unigenes were generated, including 5316 DEGs. qPCR validation showed that the expression profiles of 7 selected unigenes were consistent with those detected by RNA-seq. The diversification of the expression level of unigenes under SA induction with different time (0 h, 2 h and 8 h) reflected the complexity of the effect of SA on the transcription of *S*. *miltiorrhiza*. A number of candidate genes involved in SA signaling network in *S*. *miltiorrhiza* were discovered. In addition, several SA-responsive candidate novel genes encoding TFs, CYP450s, ABCs and proteins related to hormone crosstalk in defense have been revealed in our work. The present work also showed that SA enhanced antioxidant system in *S*. *miltiorrhiza*. All these findings are a valuable resource leading to a better understanding of the SA response network and the molecular mechanism of the effect of SA on defense and stress response at transcription level in *S*. *miltiorrhiza*. A future functional and protein interaction researches would further enable identification of essential elements in SA signaling in defense resistance of *S*. *miltiorrhiza*.

## Supporting Information

S1 Fig Distribution of predicted *S*. *miltiorrhiza* ORF length. (TIF)

S2 Fig GO classifications of the unigenes. (TIF)

S3 Fig KEGG classifications of all unigenes. (TIF)

S4 Fig Relative expression levels of 7 selected genes as detected by qPCR. (TIF)

S1 Table Primers used for gene expression level verification. (XLS)

S2 Table Summary of the statistics of RNA-seq data. (DOC)

S3 Table Size distribution of the cotigs, transcripts and unigenes assembled in *S*. *miltiorrhiza* cell cultures using the Trinity platform. (DOC)

S4 Table Summary of the statistics of unigene annotation. (DOC)

S5 Table Pearson’s Correlation Coefficient of the RNA-seq data for the samples of biological replicates for 0 hpi (T1 and T2), 2 hpi (T3, T4 and T5) and 8 hpi (T6, T7 and T8). (DOC)

S6 Table Overview of DEGs. (XLS)

S7 Table List of up, down and inconsistently regulated DEGs. (XLS)

S8 Table Statistics of GO annotation of 5316 DEGs by comparing 2 h/0 h, 8 h/0 h and 2 h/ 8 h, respectively. (XLS)

S9 Table Unigenes coding TFs. (XLS)

S10 Table List of up and down regulated candidate CYP450 genes. (XLS)

S11 Table List of identified candidate ABC transporter genes in our RNA-seq data. (XLS)

## References

[1] Kang DG, Oh H, Chung HT, Lee HS (2003) Inhibition of angiotensin converting enzyme by lithospermic acid B isolated from Radix Salviae miltiorrhiza Bunge. Phytother Res 17: 917–920. 13680824 10.1002/ptr.1250

[2] Zhou L, Zuo Z, Chow MS (2005) Danshen: an overview of its chemistry, pharmacology, pharmacokinetics, and clinical use. J Clin Pharmacol 45: 1345–1359. 16291709 10.1177/0091270005282630

[3] Yang H, Han L, Sheng T, He Q, Liang J (2006) Effects of replenishing qi, promoting blood circulation and resolving phlegm on vascular endothelial function and blood coagulation system in senile patients with hyperlipemia. J Tradit Chin Med 26: 120–124. 16817277

[4] Li YG, Song L, Liu M, Hu ZB, Wang ZT (2009) Advancement in analysis of Salviae miltiorrhizae Radix et Rhizoma (Danshen). J Chromatogr A 1216: 1941–1953. 10.1016/j.chroma.2008.12.032 19159889

[5] Raskin I (1992) Salicylate, a new plant hormone. Plant Physiol 99: 799–803. 16669002 10.1104/pp.99.3.799 PMC1080546

[6] Dong J, Wan G, Liang Z (2010) Accumulation of salicylic acid-induced phenolic compounds and raised activities of secondary metabolic and antioxidative enzymes in Salvia miltiorrhiza cell culture. J Biotechnol 148: 99–104. 10.1016/j.jbiotec.2010.05.009 20576504

[7] Hayat S, Ahmad A, Wani AS, Alyemeni MN (2014) Regulation of growth and photosynthetic parameters by salicylic acid and calcium in Brassica juncea under cadmium stress. Z Naturforsch C 69: 452–458. 10.5560/znc.2014-0036 25854765

[8] Nazar R, Umar S, Khan NA (2015) Exogenous salicylic acid improves photosynthesis and growth through increase in ascorbate-glutathione metabolism and S assimilation in mustard under salt stress. Plant Signal Behav 10: e1003751 10.1080/15592324.2014.1003751 25730495 PMC4622964

[9] Dong J, Chen C, Chen Z (2003) Expression profiles of the Arabidopsis WRKY gene superfamily during plant defense response. Plant Mol Biol 51: 21–37. 12602888 10.1023/a:1020780022549

[10] Nawrath C, Heck S, Parinthawong N, Metraux JP (2002) EDS5, an essential component of salicylic acid-dependent signaling for disease resistance in Arabidopsis, is a member of the MATE transporter family. Plant Cell 14: 275–286. 11826312 10.1105/tpc.010376 PMC150564

[11] Canet JV, Dobon A, Roig A, Tornero P (2010) Structure-function analysis of npr1 alleles in Arabidopsis reveals a role for its paralogs in the perception of salicylic acid. Plant Cell Environ 33: 1911–1922. 10.1111/j.1365-3040.2010.02194.x 20561252

[12] Fu ZQ, Yan S, Saleh A, Wang W, Ruble J, Oka N, et al (2012) NPR3 and NPR4 are receptors for the immune signal salicylic acid in plants. Nature 486: 228–232. 10.1038/nature11162 22699612 PMC3376392

[13] Zwicker S, Mast S, Stos V, Pfitzner AJ, Pfitzner UM (2007) Tobacco NIMIN2 proteins control PR gene induction through transient repression early in systemic acquired resistance. Mol Plant Pathol 8: 385–400. 10.1111/j.1364-3703.2007.00399.x 20507508

[14] Katagiri F, Lam E, Chua NH (1989) Two tobacco DNA-binding proteins with homology to the nuclear factor CREB. Nature 340: 727–730. 2528073 10.1038/340727a0

[15] Kesarwani M, Yoo J, Dong X (2007) Genetic interactions of TGA transcription factors in the regulation of pathogenesis-related genes and disease resistance in Arabidopsis. Plant Physiol 144: 336–346. 17369431 10.1104/pp.106.095299 PMC1913812

[16] Thurow C, Schiermeyer A, Krawczyk S, Butterbrodt T, Nickolov K, Gatz C (2005) Tobacco bZIP transcription factor TGA2.2 and related factor TGA2.1 have distinct roles in plant defense responses and plant development. Plant J 44: 100–113. 16167899 10.1111/j.1365-313X.2005.02513.x

[17] Wang D, Amornsiripanitch N, Dong X (2006) A genomic approach to identify regulatory nodes in the transcriptional network of systemic acquired resistance in plants. PLoS Pathog 2: e123 17096590 10.1371/journal.ppat.0020123 PMC1635530

[18] Petersen M, Brodersen P, Naested H, Andreasson E, Lindhart U, Johansen B, et al (2000) Arabidopsis map kinase 4 negatively regulates systemic acquired resistance. Cell 103: 1111–1120. 11163186 10.1016/s0092-8674(00)00213-0

[19] Frye CA, Tang D, Innes RW (2001) Negative regulation of defense responses in plants by a conserved MAPKK kinase. Proc Natl Acad Sci U S A 98: 373–378. 11114160 10.1073/pnas.98.1.373 PMC14597

[20] Zhang L, Li Y, Lu W, Meng F, Wu CA, Guo X (2012) Cotton GhMKK5 affects disease resistance, induces HR-like cell death, and reduces the tolerance to salt and drought stress in transgenic Nicotiana benthamiana. J Exp Bot 63: 3935–3951. 10.1093/jxb/ers086 22442420 PMC3388830

[21] Gao W, Sun H-X, Xiao H, Cui G, Hillwig M, Jackson A, et al (2014) Combining metabolomics and transcriptomics to characterize tanshinone biosynthesis in Salvia miltiorrhiza. BMC Genomics 15: 1–14. 24467826 10.1186/1471-2164-15-73 PMC3913955

[22] Chen S, Luo H, Li Y, Sun Y, Wu Q, Niu Y, et al (2011) 454 EST analysis detects genes putatively involved in ginsenoside biosynthesis in Panax ginseng. Plant Cell Reports 30: 1593–1601. 10.1007/s00299-011-1070-6 21484331

[23] Wenping H, Yuan Z, Jie S, Lijun Z, Zhezhi W (2011) De novo transcriptome sequencing in Salvia miltiorrhiza to identify genes involved in the biosynthesis of active ingredients. Genomics 98: 272–279. 10.1016/j.ygeno.2011.03.012 21473906

[24] Yang L, Ding G, Lin H, Cheng H, Kong Y, Wei Y, et al (2013) Transcriptome analysis of medicinal plant Salvia miltiorrhiza and identification of genes related to tanshinone biosynthesis. PLoS One 8: e80464 10.1371/journal.pone.0080464 24260395 PMC3834075

[25] Luo H, Zhu Y, Song J, Xu L, Sun C, Zhang X, et al (2014) Transcriptional data mining of Salvia miltiorrhiza in response to methyl jasmonate to examine the mechanism of bioactive compound biosynthesis and regulation. Physiol Plant 152: 241–255. 10.1111/ppl.12193 24660670

[26] Grabherr MG, Haas BJ, Yassour M, Levin JZ, Thompson DA, Amit I, et al (2011) Full-length transcriptome assembly from RNA-Seq data without a reference genome. Nat Biotechnol 29: 644–652. 10.1038/nbt.1883 21572440 PMC3571712

[27] Langmead B, Trapnell C, Pop M, Salzberg SL (2009) Ultrafast and memory-efficient alignment of short DNA sequences to the human genome. Genome Biol 10: R25 10.1186/gb-2009-10-3-r25 19261174 PMC2690996

[28] Li B, Dewey CN (2011) RSEM: accurate transcript quantification from RNA-Seq data with or without a reference genome. BMC Bioinformatics 12: 323 10.1186/1471-2105-12-323 21816040 PMC3163565

[29] Trapnell C, Williams BA, Pertea G, Mortazavi A, Kwan G, van Baren MJ, et al (2010) Transcript assembly and quantification by RNA-Seq reveals unannotated transcripts and isoform switching during cell differentiation. Nat Biotechnol 28: 511–515. 10.1038/nbt.1621 20436464 PMC3146043

[30] Anders S, Huber W (2010) Differential expression analysis for sequence count data. Genome Biol 11: R106 10.1186/gb-2010-11-10-r106 20979621 PMC3218662

[31] Benjamini Y, Drai D, Elmer G, Kafkafi N, Golani I (2001) Controlling the false discovery rate in behavior genetics research. Behav Brain Res 125: 279–284. 11682119 10.1016/s0166-4328(01)00297-2

[32] Livak KJ, Schmittgen TD (2001) Analysis of relative gene expression data using real-time quantitative PCR and the 2(-Delta Delta C(T)) Method. Methods 25: 402–408. 11846609 10.1006/meth.2001.1262

[33] Van Huylenbroeck JM, Piqueras A, Debergh PC (2000) The evolution of photosynthetic capacity and the antioxidant enzymatic system during acclimatization of micropropagated Calathea plants. Plant Sci 155: 59–66. 10773340 10.1016/s0168-9452(00)00201-6

[34] Rao MV, Paliyath G, Ormrod DP, Murr DP, Watkins CB (1997) Influence of salicylic acid on H2O2 production, oxidative stress, and H2O2-metabolizing enzymes. Salicylic acid-mediated oxidative damage requires H2O2. Plant Physiol 115: 137–149. 9306697 10.1104/pp.115.1.137 PMC158469

[35] Liu M, Qiao G, Jiang J, Yang H, Xie L, Xie J, et al (2012) Transcriptome sequencing and de novo analysis for Ma bamboo (Dendrocalamus latiflorus Munro) using the Illumina platform. PLoS One 7: e46766 10.1371/journal.pone.0046766 23056442 PMC3463524

[36] Schulze SK, Kanwar R, Golzenleuchter M, Therneau TM, Beutler AS (2012) SERE: single-parameter quality control and sample comparison for RNA-Seq. BMC Genomics 13: 524 10.1186/1471-2164-13-524 23033915 PMC3534338

[37] Dong CJ, Li L, Shang QM, Liu XY, Zhang ZG (2014) Endogenous salicylic acid accumulation is required for chilling tolerance in cucumber (Cucumis sativus L.) seedlings. Planta 240: 687–700. 10.1007/s00425-014-2115-1 25034826

[38] Wang M, Zhang Y, Wang J, Wu X, Guo X (2007) A novel MAP kinase gene in cotton (Gossypium hirsutum L.), GhMAPK, is involved in response to diverse environmental stresses. J Biochem Mol Biol 40: 325–332. 17562283 10.5483/bmbrep.2007.40.3.325

[39] Melvin P, Prabhu SA, Veena M, Shailasree S, Petersen M, Mundy J, et al (2015) The pearl millet mitogen-activated protein kinase PgMPK4 is involved in responses to downy mildew infection and in jasmonic- and salicylic acid-mediated defense. Plant Mol Biol 87: 287–302. 10.1007/s11103-014-0276-8 25527312

[40] Xia XJ, Zhou YH, Shi K, Zhou J, Foyer CH, Yu JQ (2015) Interplay between reactive oxygen species and hormones in the control of plant development and stress tolerance. J Exp Bot 66: 2839–2856. 10.1093/jxb/erv089 25788732

[41] Zhu S, Tang S, Tang Q, Liu T (2014) Genome-wide transcriptional changes of ramie (Boehmeria nivea L. Gaud) in response to root-lesion nematode infection. Gene 552: 67–74. 10.1016/j.gene.2014.09.014 25218245

[42] Metraux JP, Signer H, Ryals J, Ward E, Wyss-Benz M, Gaudin J, et al (1990) Increase in salicylic Acid at the onset of systemic acquired resistance in cucumber. Science 250: 1004–1006. 17746926 10.1126/science.250.4983.1004

[43] Herrera-Vasquez A, Salinas P, Holuigue L (2015) Salicylic acid and reactive oxygen species interplay in the transcriptional control of defense genes expression. Front Plant Sci 6: 171 10.3389/fpls.2015.00171 25852720 PMC4365548

[44] Malamy J, Carr JP, Klessig DF, Raskin I (1990) Salicylic Acid: a likely endogenous signal in the resistance response of tobacco to viral infection. Science 250: 1002–1004. 17746925 10.1126/science.250.4983.1002

[45] Zhang Y, Tessaro MJ, Lassner M, Li X (2003) Knockout analysis of Arabidopsis transcription factors TGA2, TGA5, and TGA6 reveals their redundant and essential roles in systemic acquired resistance. Plant Cell 15: 2647–2653. 14576289 10.1105/tpc.014894 PMC280568

[46] An C, Mou Z (2011) Salicylic acid and its function in plant immunity. J Integr Plant Biol 53: 412–428. 10.1111/j.1744-7909.2011.01043.x 21535470

[47] Zhang Y, Cheng YT, Qu N, Zhao Q, Bi D, Li X (2006) Negative regulation of defense responses in Arabidopsis by two NPR1 paralogs. Plant J 48: 647–656. 17076807 10.1111/j.1365-313X.2006.02903.x

[48] Liu G, Holub EB, Alonso JM, Ecker JR, Fobert PR (2005) An Arabidopsis NPR1-like gene, NPR4, is required for disease resistance. Plant J 41: 304–318. 15634206 10.1111/j.1365-313X.2004.02296.x

[49] Despres C, DeLong C, Glaze S, Liu E, Fobert PR (2000) The Arabidopsis NPR1/NIM1 protein enhances the DNA binding activity of a subgroup of the TGA family of bZIP transcription factors. Plant Cell 12: 279–290. 10662863 PMC139764

[50] Gao QM, Venugopal S, Navarre D, Kachroo A (2011) Low oleic acid-derived repression of jasmonic acid-inducible defense responses requires the WRKY50 and WRKY51 proteins. Plant Physiol 155: 464–476. 10.1104/pp.110.166876 21030507 PMC3075765

[51] Encinas-Villarejo S, Maldonado AM, Amil-Ruiz F, de los Santos B, Romero F, Pliego-Alfaro F, et al (2009) Evidence for a positive regulatory role of strawberry (Fragaria x ananassa) Fa WRKY1 and Arabidopsis At WRKY75 proteins in resistance. J Exp Bot 60: 3043–3065. 10.1093/jxb/erp152 19470657

[52] Li J, Brader G, Palva ET (2004) The WRKY70 transcription factor: a node of convergence for jasmonate-mediated and salicylate-mediated signals in plant defense. Plant Cell 16: 319–331. 14742872 10.1105/tpc.016980 PMC341906

[53] Wu KL, Guo ZJ, Wang HH, Li J (2005) The WRKY family of transcription factors in rice and Arabidopsis and their origins. DNA Res 12: 9–26. 16106749 10.1093/dnares/12.1.9

[54] Chen C, Chen Z (2002) Potentiation of developmentally regulated plant defense response by AtWRKY18, a pathogen-induced Arabidopsis transcription factor. Plant Physiol 129: 706–716. 12068113 10.1104/pp.001057 PMC161695

[55] Journot-Catalino N, Somssich IE, Roby D, Kroj T (2006) The transcription factors WRKY11 and WRKY17 act as negative regulators of basal resistance in Arabidopsis thaliana. Plant Cell 18: 3289–3302. 17114354 10.1105/tpc.106.044149 PMC1693958

[56] Xing Y, Chen WH, Jia W, Zhang J (2015) Mitogen-activated protein kinase kinase 5 (MKK5)-mediated signalling cascade regulates expression of iron superoxide dismutase gene in Arabidopsis under salinity stress. J Exp Bot. 10.1093/jxb/erv305 PMC4566985 26136265

[57] Doczi R, Brader G, Pettko-Szandtner A, Rajh I, Djamei A, Pitzschke A, et al (2007) The Arabidopsis mitogen-activated protein kinase kinase MKK3 is upstream of group C mitogen-activated protein kinases and participates in pathogen signaling. Plant Cell 19: 3266–3279. 17933903 10.1105/tpc.106.050039 PMC2174707

[58] Chaturvedi R, Krothapalli K, Makandar R, Nandi A, Sparks AA, Roth MR, et al (2008) Plastid omega3-fatty acid desaturase-dependent accumulation of a systemic acquired resistance inducing activity in petiole exudates of Arabidopsis thaliana is independent of jasmonic acid. Plant J 54: 106–117. 18088304 10.1111/j.1365-313X.2007.03400.x

[59] Manosalva PM, Park SW, Forouhar F, Tong L, Fry WE, Klessig DF (2010) Methyl esterase 1 (StMES1) is required for systemic acquired resistance in potato. Mol Plant Microbe Interact 23: 1151–1163. 10.1094/MPMI-23-9-1151 20687805

[60] Kumar D, Klessig DF (2003) High-affinity salicylic acid-binding protein 2 is required for plant innate immunity and has salicylic acid-stimulated lipase activity. Proc Natl Acad Sci U S A 100: 16101–16106. 14673096 10.1073/pnas.0307162100 PMC307699

[61] Shah J, Zeier J (2013) Long-distance communication and signal amplification in systemic acquired resistance. Front Plant Sci 4: 30 10.3389/fpls.2013.00030 23440336 PMC3579191

[62] Ji LH, Ding SW (2001) The suppressor of transgene RNA silencing encoded by Cucumber mosaic virus interferes with salicylic acid-mediated virus resistance. Mol Plant Microbe Interact 14: 715–724. 11386367 10.1094/MPMI.2001.14.6.715

[63] Carr JP, Lewsey MG, Palukaitis P (2010) Signaling in induced resistance. Adv Virus Res 76: 57–121. 10.1016/S0065-3527(10)76003-6 20965072

[64] Wong CE, Carson RA, Carr JP (2002) Chemically induced virus resistance in Arabidopsis thaliana is independent of pathogenesis-related protein expression and the NPR1 gene. Mol Plant Microbe Interact 15: 75–81. 11858174 10.1094/MPMI.2002.15.1.75

[65] Jaubert M, Bhattacharjee S, Mello AF, Perry KL, Moffett P (2011) ARGONAUTE2 mediates RNA-silencing antiviral defenses against Potato virus X in Arabidopsis. Plant Physiol 156: 1556–1564. 10.1104/pp.111.178012 21576511 PMC3135937

[66] Yu D, Fan B, MacFarlane SA, Chen Z (2003) Analysis of the involvement of an inducible Arabidopsis RNA-dependent RNA polymerase in antiviral defense. Mol Plant Microbe Interact 16: 206–216. 12650452 10.1094/MPMI.2003.16.3.206

[67] Quan LJ, Zhang B, Shi WW, Li HY (2008) Hydrogen peroxide in plants: a versatile molecule of the reactive oxygen species network. J Integr Plant Biol 50: 2–18. 10.1111/j.1744-7909.2007.00599.x 18666947

[68] Asada K (2006) Production and scavenging of reactive oxygen species in chloroplasts and their functions. Plant Physiol 141: 391–396. 16760493 10.1104/pp.106.082040 PMC1475469

[69] Foyer CH, Noctor G (2011) Ascorbate and glutathione: the heart of the redox hub. Plant Physiol 155: 2–18. 10.1104/pp.110.167569 21205630 PMC3075780

[70] Bolwell GP, Blee KA, Butt VS, Davies DR, Gardner SL, Gerrish C, et al (1999) Recent advances in understanding the origin of the apoplastic oxidative burst in plant cells. Free Radic Res 31 Suppl: S137–145. 10694052 10.1080/10715769900301431

[71] Alscher RG, Erturk N, Heath LS (2002) Role of superoxide dismutases (SODs) in controlling oxidative stress in plants. J Exp Bot 53: 1331–1341. 11997379

[72] Gleason JE, Li CX, Odeh HM, Culotta VC (2014) Species-specific activation of Cu/Zn SOD by its CCS copper chaperone in the pathogenic yeast Candida albicans. J Biol Inorg Chem 19: 595–603. 10.1007/s00775-013-1045-x 24043471 PMC3956745

[73] Abdel-Ghany SE, Burkhead JL, Gogolin KA, Andres-Colas N, Bodecker JR, Puig S, et al (2005) AtCCS is a functional homolog of the yeast copper chaperone Ccs1/Lys7. FEBS Lett 579: 2307–2312. 15848163 10.1016/j.febslet.2005.03.025

[74] Mutlu S, Atici Ö, Nalbantoglu B (2009) Effects of salicylic acid and salinity on apoplastic antioxidant enzymes in two wheat cultivars differing in salt tolerance. Biologia Plantarum 53: 334–338.

[75] Li T, Hu Y, Du X, Tang H, Shen C, Wu J (2014) Salicylic acid alleviates the adverse effects of salt stress in Torreya grandis cv. Merrillii seedlings by activating photosynthesis and enhancing antioxidant systems. PLoS One 9: e109492 10.1371/journal.pone.0109492 25302987 PMC4193794

[76] Schmidt M, Dringen R (2012) Glutathione (GSH) Synthesis and Metabolism In: Choi I-Y, Gruetter R, editors. Neural Metabolism In Vivo: Springer US pp. 1029–1050.

[77] Commandeur JN, Stijntjes GJ, Vermeulen NP (1995) Enzymes and transport systems involved in the formation and disposition of glutathione S-conjugates. Role in bioactivation and detoxication mechanisms of xenobiotics. Pharmacol Rev 47: 271–330. 7568330

[78] Mateo A, Funck D, Muhlenbock P, Kular B, Mullineaux PM, Karpinski S (2006) Controlled levels of salicylic acid are required for optimal photosynthesis and redox homeostasis. J Exp Bot 57: 1795–1807. 16698814 10.1093/jxb/erj196

[79] Han Y, Chaouch S, Mhamdi A, Queval G, Zechmann B, Noctor G (2013) Functional analysis of Arabidopsis mutants points to novel roles for glutathione in coupling H(2)O(2) to activation of salicylic acid accumulation and signaling. Antioxid Redox Signal 18: 2106–2121. 10.1089/ars.2012.5052 23148658 PMC3629853

[80] Dubreuil-Maurizi C, Vitecek J, Marty L, Branciard L, Frettinger P, Wendehenne D, et al (2011) Glutathione deficiency of the Arabidopsis mutant pad2-1 affects oxidative stress-related events, defense gene expression, and the hypersensitive response. Plant Physiol 157: 2000–2012. 10.1104/pp.111.182667 22007023 PMC3327178

[81] Marrs KA (1996) The functions and regulation of glutathione s-transferases in plants. Annu Rev Plant Physiol Plant Mol Biol 47: 127–158. 15012285 10.1146/annurev.arplant.47.1.127

[82] Vlot AC, Dempsey DA, Klessig DF (2009) Salicylic Acid, a multifaceted hormone to combat disease. Annu Rev Phytopathol 47: 177–206. 10.1146/annurev.phyto.050908.135202 19400653

[83] Yang YX, Ahammed GJ, Wu C, Fan SY, Zhou YH (2015) Crosstalk among Jasmonate, Salicylate and Ethylene Signaling Pathways in Plant Disease and Immune Responses. Curr Protein Pept Sci 16: 450–461. 25824390 10.2174/1389203716666150330141638

[84] Leon-Reyes A, Van der Does D, De Lange ES, Delker C, Wasternack C, Van Wees SC, et al (2010) Salicylate-mediated suppression of jasmonate-responsive gene expression in Arabidopsis is targeted downstream of the jasmonate biosynthesis pathway. Planta 232: 1423–1432. 10.1007/s00425-010-1265-z 20839007 PMC2957573

[85] Mitsuhara I, Iwai T, Seo S, Yanagawa Y, Kawahigasi H, Hirose S, et al (2008) Characteristic expression of twelve rice PR1 family genes in response to pathogen infection, wounding, and defense-related signal compounds (121/180). Mol Genet Genomics 279: 415–427. 10.1007/s00438-008-0322-9 18247056 PMC2270915

[86] Ndamukong I, Abdallat AA, Thurow C, Fode B, Zander M, Weigel R, et al (2007) SA-inducible Arabidopsis glutaredoxin interacts with TGA factors and suppresses JA-responsive PDF1.2 transcription. Plant J 50: 128–139. 17397508 10.1111/j.1365-313X.2007.03039.x

[87] Spoel SH, Koornneef A, Claessens SM, Korzelius JP, Van Pelt JA, Mueller MJ, et al (2003) NPR1 modulates cross-talk between salicylate- and jasmonate-dependent defense pathways through a novel function in the cytosol. Plant Cell 15: 760–770. 12615947 10.1105/tpc.009159 PMC150028

[88] Grefen C, Stadele K, Ruzicka K, Obrdlik P, Harter K, Horak J (2008) Subcellular localization and in vivo interactions of the Arabidopsis thaliana ethylene receptor family members. Mol Plant 1: 308–320. 10.1093/mp/ssm015 19825542

[89] Berrocal-Lobo M, Molina A (2004) Ethylene response factor 1 mediates Arabidopsis resistance to the soilborne fungus Fusarium oxysporum. Mol Plant Microbe Interact 17: 763–770. 15242170 10.1094/MPMI.2004.17.7.763

[90] de Torres Zabala M, Bennett MH, Truman WH, Grant MR (2009) Antagonism between salicylic and abscisic acid reflects early host-pathogen conflict and moulds plant defence responses. Plant J 59: 375–386. 10.1111/j.1365-313X.2009.03875.x 19392690

[91] Raghavendra AS, Gonugunta VK, Christmann A, Grill E (2010) ABA perception and signalling. Trends Plant Sci 15: 395–401. 10.1016/j.tplants.2010.04.006 20493758

[92] Wang D, Pajerowska-Mukhtar K, Culler AH, Dong X (2007) Salicylic acid inhibits pathogen growth in plants through repression of the auxin signaling pathway. Curr Biol 17: 1784–1790. 17919906 10.1016/j.cub.2007.09.025

[93] To JP, Deruere J, Maxwell BB, Morris VF, Hutchison CE, Ferreira FJ, et al (2007) Cytokinin regulates type-A Arabidopsis Response Regulator activity and protein stability via two-component phosphorelay. Plant Cell 19: 3901–3914. 18065689 10.1105/tpc.107.052662 PMC2217641

[94] Daviere JM, Achard P (2013) Gibberellin signaling in plants. Development 140: 1147–1151. 10.1242/dev.087650 23444347

[95] Balazadeh S, Riano-Pachon DM, Mueller-Roeber B (2008) Transcription factors regulating leaf senescence in Arabidopsis thaliana. Plant Biol (Stuttg) 10 Suppl 1: 63–75. 18721312 10.1111/j.1438-8677.2008.00088.x

[96] Chen YN, Slabaugh E, Brandizzi F (2008) Membrane-tethered transcription factors in Arabidopsis thaliana: novel regulators in stress response and development. Curr Opin Plant Biol 11: 695–701. 10.1016/j.pbi.2008.10.005 19019722

[97] Ma HS, Liang D, Shuai P, Xia XL, Yin WL (2010) The salt- and drought-inducible poplar GRAS protein SCL7 confers salt and drought tolerance in Arabidopsis thaliana. J Exp Bot 61: 4011–4019. 10.1093/jxb/erq217 20616154 PMC2935874

[98] Sun L, Huang L, Hong Y, Zhang H, Song F, Li D (2015) Comprehensive analysis suggests overlapping expression of rice ONAC transcription factors in abiotic and biotic stress responses. Int J Mol Sci 16: 4306–4326. 10.3390/ijms16024306 25690040 PMC4346958

[99] Harris JC, Hrmova M, Lopato S, Langridge P (2011) Modulation of plant growth by HD-Zip class I and II transcription factors in response to environmental stimuli. New Phytol 190: 823–837. 10.1111/j.1469-8137.2011.03733.x 21517872

[100] Zhang Y, Mayba O, Pfeiffer A, Shi H, Tepperman JM, Speed TP, et al (2013) A quartet of PIF bHLH factors provides a transcriptionally centered signaling hub that regulates seedling morphogenesis through differential expression-patterning of shared target genes in Arabidopsis. PLoS Genet 9: e1003244 10.1371/journal.pgen.1003244 23382695 PMC3561105

[101] Cote CL, Boileau F, Roy V, Ouellet M, Levasseur C, Morency MJ, et al (2010) Gene family structure, expression and functional analysis of HD-Zip III genes in angiosperm and gymnosperm forest trees. BMC Plant Biol 10: 273 10.1186/1471-2229-10-273 21143995 PMC3017839

[102] Li J, Han Y, Liu L, Chen Y, Du Y, Zhang J, et al (2015) qRT9, a quantitative trait locus controlling root thickness and root length in upland rice. J Exp Bot 66: 2723–2732. 10.1093/jxb/erv076 25769309

[103] Liu C, Mao B, Ou S, Wang W, Liu L, Wu Y, et al (2014) OsbZIP71, a bZIP transcription factor, confers salinity and drought tolerance in rice. Plant Mol Biol 84: 19–36. 10.1007/s11103-013-0115-3 23918260

[104] Llorca CM, Potschin M, Zentgraf U (2014) bZIPs and WRKYs: two large transcription factor families executing two different functional strategies. Front Plant Sci 5: 169 10.3389/fpls.2014.00169 24817872 PMC4012195

[105] Gao C, Li P, Song A, Wang H, Wang Y, Ren L, et al (2015) Isolation and characterization of six AP2/ERF transcription factor genes in Chrysanthemum nankingense. Int J Mol Sci 16: 2052–2065. 10.3390/ijms16012052 25607731 PMC4307348

[106] Shi G, Guo X, Guo J, Liu L, Hua J (2015) Analyzing serial cDNA libraries revealed reactive oxygen species and gibberellins signaling pathways in the salt response of Upland cotton (Gossypium hirsutum L.). Plant Cell Reports 34: 1005–1023. 10.1007/s00299-015-1761-5 25700980

[107] Qi X, Bakht S, Qin B, Leggett M, Hemmings A, Mellon F, et al (2006) A different function for a member of an ancient and highly conserved cytochrome P450 family: from essential sterols to plant defense. Proc Natl Acad Sci U S A 103: 18848–18853. 17124172 10.1073/pnas.0607849103 PMC1656972

[108] Irmisch S, Clavijo McCormick A, Gunther J, Schmidt A, Boeckler GA, Gershenzon J, et al (2014) Herbivore-induced poplar cytochrome P450 enzymes of the CYP71 family convert aldoximes to nitriles which repel a generalist caterpillar. Plant J 80: 1095–1107. 10.1111/tpj.12711 25335755

[109] Theodoulou FL (2000) Plant ABC transporters. Biochim Biophys Acta 1465: 79–103. 10748248 10.1016/s0005-2736(00)00132-2

[110] Krattinger SG, Lagudah ES, Spielmeyer W, Singh RP, Huerta-Espino J, McFadden H, et al (2009) A putative ABC transporter confers durable resistance to multiple fungal pathogens in wheat. Science 323: 1360–1363. 10.1126/science.1166453 19229000

[111] Wang X, Liu Y, Chen L, Zhao D, Zhang Z (2013) Wheat resistome in response to barley yellow dwarf virus infection. Funct Integr Genomics 13: 155–165. 10.1007/s10142-013-0309-4 23417744

[112] Remy E, Cabrito TR, Baster P, Batista RA, Teixeira MC, Friml J, et al (2013) A major facilitator superfamily transporter plays a dual role in polar auxin transport and drought stress tolerance in Arabidopsis. Plant Cell 25: 901–926. 10.1105/tpc.113.110353 23524662 PMC3634696

[113] Brunetti P, Zanella L, De Paolis A, Di Litta D, Cecchetti V, Falasca G, et al (2015) Cadmium-inducible expression of the ABC-type transporter AtABCC3 increases phytochelatin-mediated cadmium tolerance in Arabidopsis. J Exp Bot 66: 3815–3829. 10.1093/jxb/erv185 25900618 PMC4473984

[114] Baron KN, Schroeder DF, Stasolla C (2012) Transcriptional response of abscisic acid (ABA) metabolism and transport to cold and heat stress applied at the reproductive stage of development in Arabidopsis thaliana. Plant Sci 188–189: 48–59. 10.1016/j.plantsci.2012.03.001 22525244

