# Supplementary figures and images for: Transcriptome Sequencing in Response to Salicylic Acid in Salvia miltiorrhiza
Source: PLoS One. 2016 Jan 25;11(1):e0147849. doi: 10.1371/journal.pone.0147849 (PMC4726470; doi:10.1371/journal.pone.0147849)

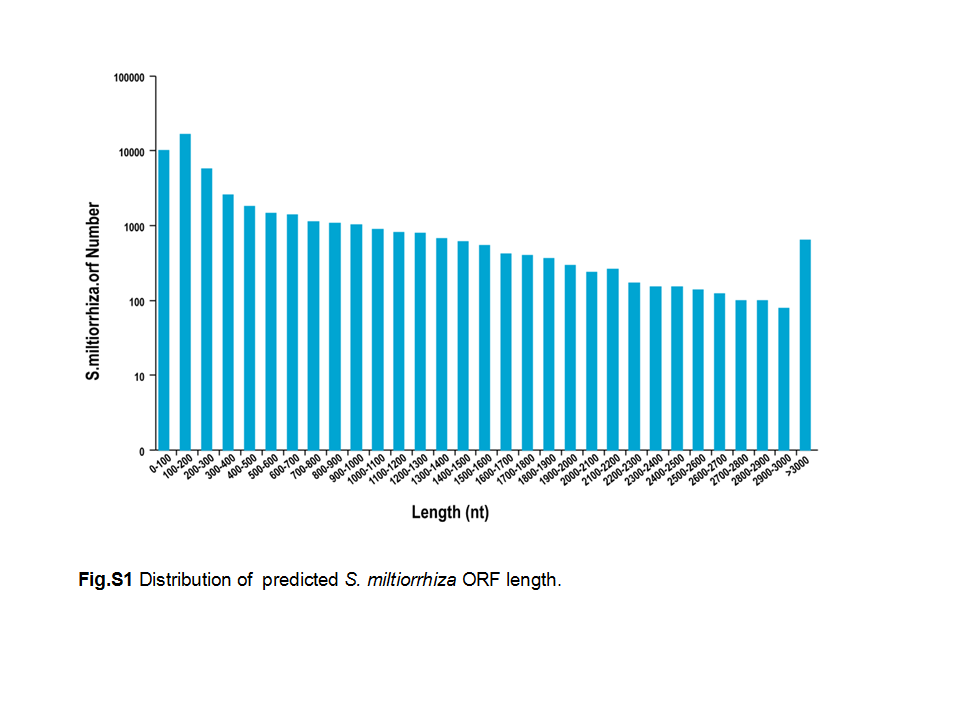

Supplement: S1 Fig — (TIF) [file pone.0147849.s001.tif]

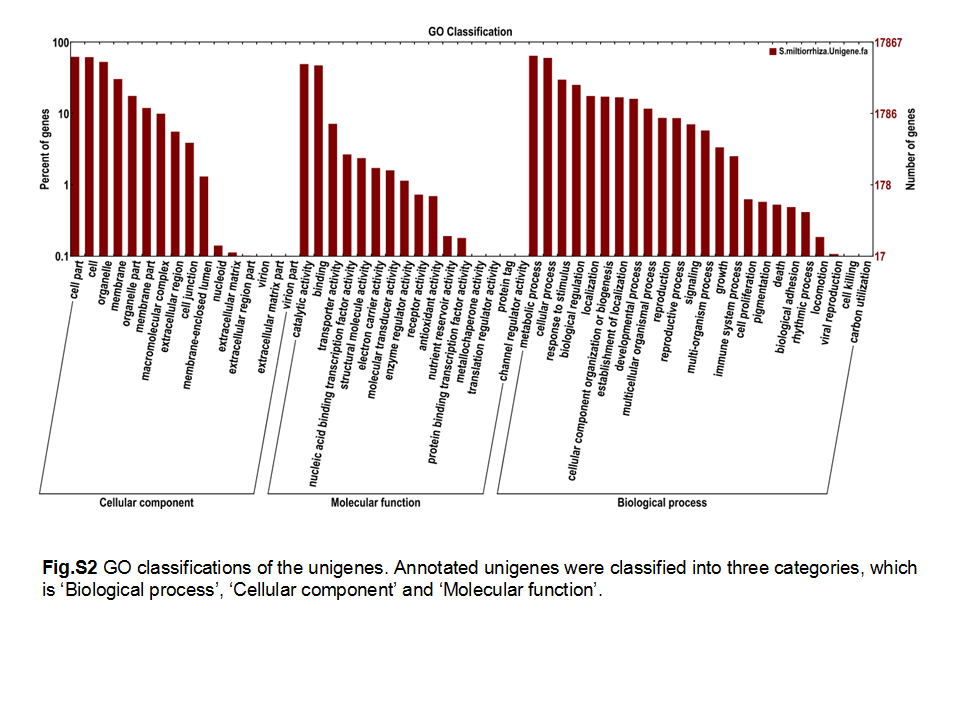

Supplement: S2 Fig — (TIF) [file pone.0147849.s002.tif]

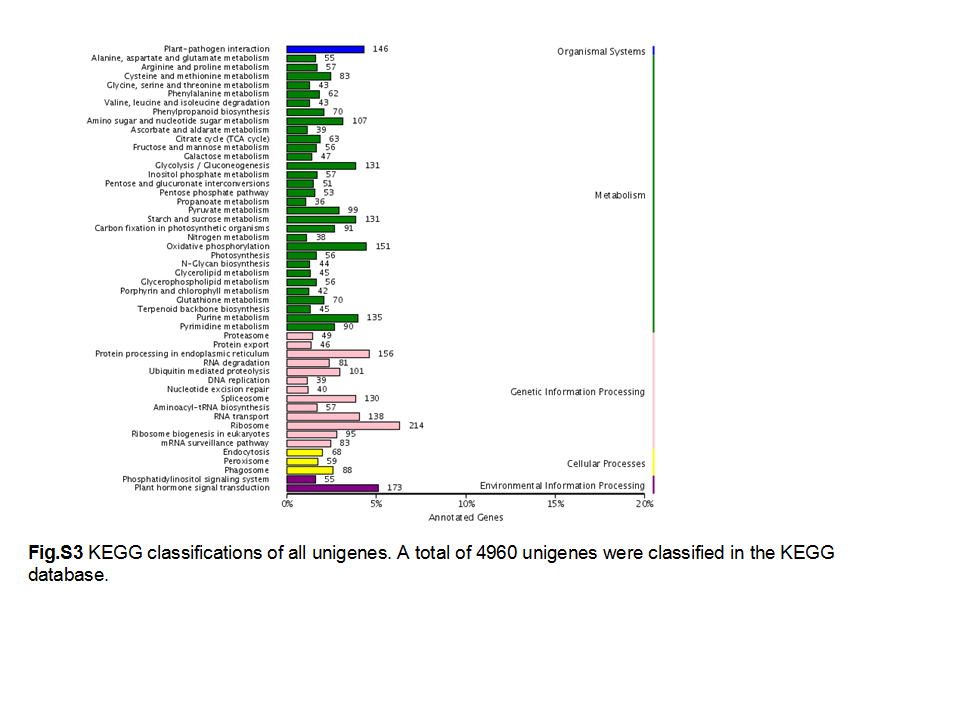

Supplement: S3 Fig — (TIF) [file pone.0147849.s003.tif]

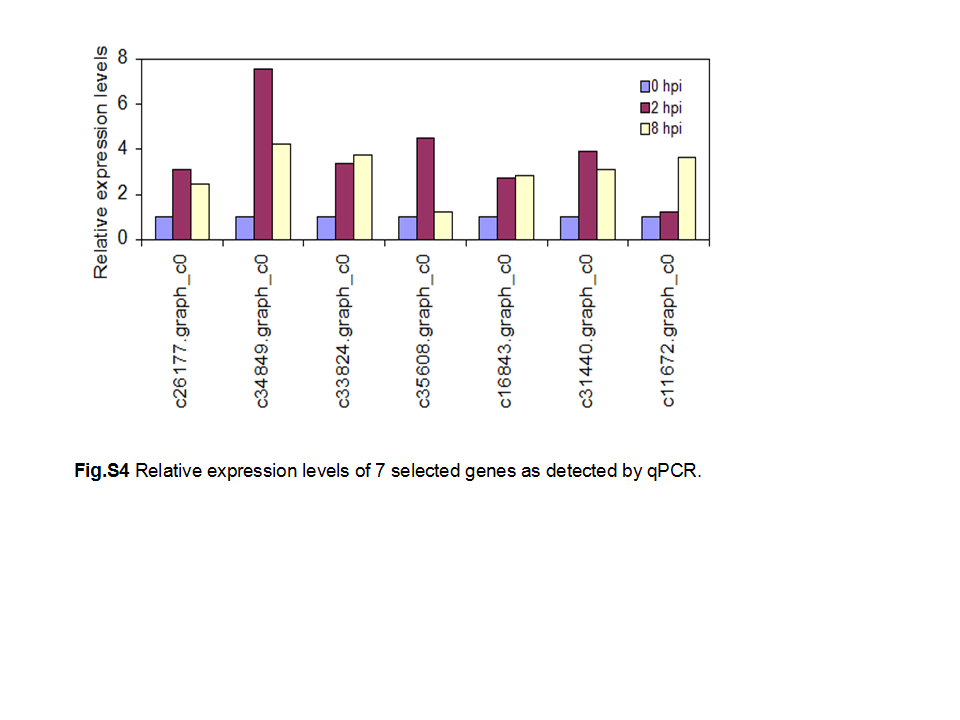

Supplement: S4 Fig — (TIF) [file pone.0147849.s004.tif]
