# Supplementary material for: Transcriptome Sequencing in Response to Salicylic Acid in Salvia miltiorrhiza
Source: PLoS One. 2016 Jan 25;11(1):e0147849. doi: 10.1371/journal.pone.0147849 (PMC4726470; doi:10.1371/journal.pone.0147849)
Supplement: S2 Table — (DOC) [file pone.0147849.s006.doc]

**Table S2. Summary of the statistics of RNA-seq data. The sample type, sample ID, read number, base number, GC content and Q30(％) are shown.**

| **Sample Type** | **Sample ID** | **Read Number** | **Base Number** | **GC Content** | **%≥Q30** |
| --- | --- | --- | --- | --- | --- |
| CK | T1 | 16,930,116 | 3,419,495,711 | 49.73% | 87.91% |
| CK2 | T2 | 16,304,238 | 3,293,099,648 | 49.78% | 87.60% |
| SA2-1 | T3 | 17,092,890 | 3,452,363,358 | 50.38% | 87.31% |
| SA2-2 | T4 | 16,519,814 | 3,336,556,340 | 50.56% | 86.65% |
| SA2-3 | T5 | 14,846,248 | 2,998,365,181 | 50.36% | 87.04% |
| SA8-1 | T6 | 17,074,504 | 3,440,916,881 | 49.03% | 87.56% |
| SA8-2 | T7 | 15,929,201 | 3,217,116,423 | 49.87% | 87.60% |
| SA8-3 | T8 | 15,182,299 | 3,066,223,529 | 50.06% | 86.71% |
| IlluminaHiseq 2500 platform was used to sequence the transcriptome for these samples and 100 bp paired-end reads were generated for these cDNA libraries. | | | | | |
