# Supplementary material for: Transcriptome Sequencing in Response to Salicylic Acid in Salvia miltiorrhiza
Source: PLoS One. 2016 Jan 25;11(1):e0147849. doi: 10.1371/journal.pone.0147849 (PMC4726470; doi:10.1371/journal.pone.0147849)
Supplement: S3 Table — (DOC) [file pone.0147849.s007.doc]

**Table S3.** **Size distribution of the cotigs, transcripts and unigenes assembled in *S. miltiorrhiza* cell cultures using the Trinity platform.**

| **Length Range(bp)** | **Contig** | **Transcript** | **Unigene** |
| --- | --- | --- | --- |
| 200-300 | 14 521 250(99.71%) | 20 163(16.13%) | 15 863(31.24%) |
| 300-500 | 15 253(0.10%) | 18 390(14.71%) | 11 665(22.97%) |
| 500-1000 | 11 931(0.08%) | 23 687(18.95%) | 9 341(18.40%) |
| 1000-2000 | 9 476(0.07%) | 33 541(26.83%) | 8 203(16.15%) |
| >2000 | 5 400(0.04%) | 29 243(23.39%) | 5 706(11.24%) |
| Total Number | 14 563 310 | 125 024 | 50 778 |
| Total Length | 555 086 854 | 168 790 298 | 44 113 292 |
| N50 Length | 38 | 2 105 | 1 618 |
| Mean Length | 38.12 | 1 350.06 | 868.75 |
