# Supplementary material for: Transcriptome Sequencing in Response to Salicylic Acid in Salvia miltiorrhiza
Source: PLoS One. 2016 Jan 25;11(1):e0147849. doi: 10.1371/journal.pone.0147849 (PMC4726470; doi:10.1371/journal.pone.0147849)
Supplement: S4 Table — (DOC) [file pone.0147849.s008.doc]

**Table S4. Summary of the statistics of unigene annotation.**

| **Annotated databases** | **Unigene** | **≥300nt** | **≥1000nt** |
| --- | --- | --- | --- |
| COG | 7,881 | 7,456 | 5,579 |
| GO | 17,867 | 16,013 | 10,391 |
| KEGG | 4,960 | 4,545 | 3,148 |
| Swiss-Prot | 19,261 | 17,482 | 11,516 |
| nr | 24,130 | 21,384 | 13,086 |
| All | 24,181 | 21,419 | 13,091 |
