# Supplementary material for: Transcriptome Sequencing in Response to Salicylic Acid in Salvia miltiorrhiza
Source: PLoS One. 2016 Jan 25;11(1):e0147849. doi: 10.1371/journal.pone.0147849 (PMC4726470; doi:10.1371/journal.pone.0147849)
Supplement: S5 Table — (DOC) [file pone.0147849.s009.doc]

**Table S5. Pearson’s Correlation Coefficient of the RNA-seq data for the samples of biological replicates for 0 hpi (T1 and T2), 2 hpi (T3, T4 and T5) and 8 hpi (T6, T7 and T8).**

| **Sample 1** | **Sample 2** | **r^2** |
| --- | --- | --- |
| T1 | T2 | 0.9107 |
| T3 | T4 | 0.9955 |
| T3 | T5 | 0.9972 |
| T6 | T7 | 0.9537 |
| T6 | T8 | 0.9502 |
